# Structural insights into the mechanism of protein transport by the Type 9 Secretion System translocon

**DOI:** 10.1038/s41564-024-01644-7

**Published:** 2024-03-27

**Authors:** Frédéric Lauber, Justin C. Deme, Xiaolong Liu, Andreas Kjær, Helen L. Miller, Felicity Alcock, Susan M. Lea, Ben C. Berks

**Affiliations:** 1 Department of Biochemistry, University of Oxford, Oxford, UK; 2 Center for Structural Biology, Center for Cancer Research, National Cancer Institute, Frederick, MD USA; 3 Sir William Dunn School of Pathology, University of Oxford, Oxford, UK; 4 The Central Oxford Structural Molecular Imaging Centre (COSMIC), University of Oxford, Oxford, UK; 5 Biological Physics Research Group, Department of Physics, University of Oxford, Oxford, UK; 6 Present Address: de Duve Institute, Université Catholique de Louvain, Brussels, Belgium; 7 Present Address: Newcastle University Biosciences Institute, Newcastle University, Newcastle, UK

**Keywords:** Cellular microbiology, Bacterial secretion, Bacterial structural biology

## Abstract

Secretion systems are protein export machines that enable bacteria to exploit their environment through the release of protein effectors. The Type 9 Secretion System (T9SS) is responsible for protein export across the outer membrane (OM) of bacteria of the phylum Bacteroidota. Here we trap the T9SS of *Flavobacterium johnsoniae* in the process of substrate transport by disrupting the T9SS motor complex. Cryo-EM analysis of purified substrate-bound T9SS translocons reveals an extended translocon structure in which the previously described translocon core is augmented by a periplasmic structure incorporating the proteins SprE, PorD and a homologue of the canonical periplasmic chaperone Skp. Substrate proteins bind to the extracellular loops of a carrier protein within the translocon pore. As transport intermediates accumulate on the translocon when energetic input is removed, we deduce that release of the substrate–carrier protein complex from the translocon is the energy-requiring step in T9SS transport.

## Abstract (web summary)

Cryo-EM analysis of the substrate-bound T9SS from *Flavobacterium johnsoniae* reveals an extended translocon complex and provides insight into protein secretion.

## Main

Substrates of the Type 9 Secretion System (T9SS)^1–3^ are targeted to the T9SS translocon by a C-terminal domain (CTD)^4,5^ which adopts a 7-stranded β-sandwich structure^6–9^. CTDs can be divided into two distinct classes referred to as Type A and Type B^1,5,10^. Substrate proteins with a Type A CTD are carried from the translocon by the OM protein PorV and are subsequently either released into the cellular environment or linked to a cell surface lipopolysaccharide molecule^11,12^. Substrates with a Type B CTD leave the translocon bound to alternative carrier proteins which then anchor them to the cell surface^13,14^.

Protein transport through the T9SS translocon is powered by an energy chain. In gliding Bacteroidota the same energy chain also propels the cell surface adhesins that mediate gliding motility^15,16^. In the energy chain, an inner membrane (IM)-anchored periplasm-spanning motor complex formed by the proteins GldLM (*F. johnsoniae* nomenclature) is first used to transfer the energy of the protonmotive force (PMF) across the periplasm to a structure at the inner face of the OM^17–22^, which we term the Hub complex. The Hub complex then distributes energy to both the T9SS translocon and the gliding motility system.

Characterization of the T9SS translocon from the gliding bacterium *F. johnsoniae* shows that it is composed of a 36-strand transmembrane β-barrel protein SprA and a peptidyl-prolyl *cis–trans* isomerase (PPI) subunit of unknown function^23^. The SprA barrel pore is capped on the extracellular side but has a lateral opening to the external membrane surface. Two distinct forms of the translocon have been isolated, containing either the Type A substrate carrier protein PorV (PorV complex) or a protein termed Plug (Plug complex) which seals the quiescent translocon. PorV binds across the lateral opening of SprA in such a way that three of the extracellular loops of PorV protrude into the SprA pore. On the basis of these structures, it has been suggested that substrate molecules enter the periplasmic end of the SprA pore, bind to the PorV loops and then exit the translocon through the lateral opening in complex with PorV^23^. However, in the absence of substrate-bound structures, this provisional mechanism remains speculative.

Here we show that substrates can be trapped on the translocon complex by blocking power input to the T9SS. Structural analysis of these transport intermediates confirms that substrate proteins bind inside the translocon pore in contact with PorV and reveals how the translocon recognizes the non-identical CTDs found on different substrate proteins. Unexpectedly, we find that the previously described T9SS translocon is only the nucleus of a much larger structure that we name the Extended Translocon.

## Results

### Isolation of a substrate-bound Extended Translocon

**Fig. 1:**
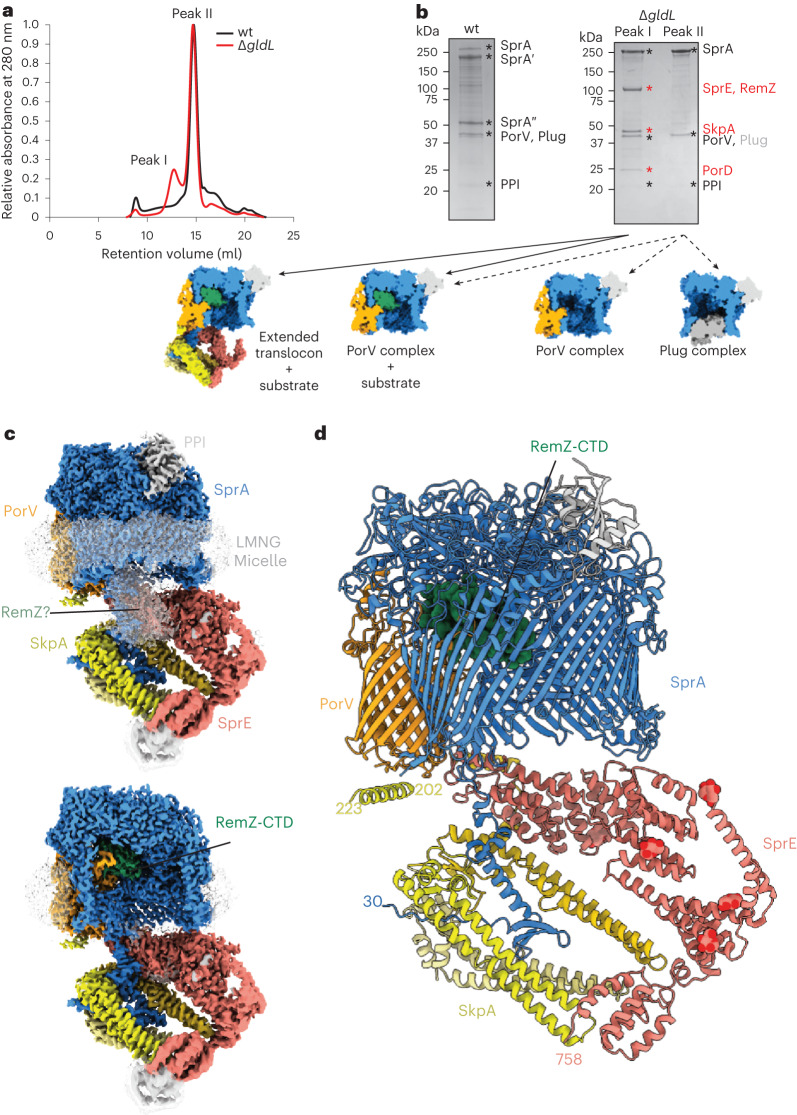
Purification of a substrate-bound extended T9SS translocon complex. **a**, Size exclusion chromatography profiles of Twin-strep-tagged SprA complexes purified by Streptactin-affinity chromatography from wild-type (wt; black) and Δ*gldL* mutant (red) strains. **b**, Top: Coomassie-stained sodium dodecyl-sulfate polyacrylamide gel electrophoresis (SDS–PAGE) gels of the peak fractions from **a**. Band identities were assigned on the basis of peptide fingerprinting. SprAʹ and SprAʺ arise from proteolytic clipping in the loop between strands 24 and 25 of the SprA barrel^23^. Proteins detected in all samples are labelled in black. Protein bands only visible in Peak I of the Δ*gldL* purification are labelled in red. Only trace levels of Plug (grey) were detected in Peak I. Similar data were obtained from two independent preparations. Bottom: the different classes of SprA complexes identified in peaks I (solid arrows) and II (dashed arrows) by cryo-EM. **c**, Cryo-EM volume of the substrate-bound Extended Translocon complex coloured by subunit. The detergent micelle and other unmodelled densities are shown in white at a lower contour level. The lower panel is cut through to show the position of the substrate CTD. **d**, Atomic model of the Extended Translocon shown in cartoon representation with the substrate CTD in spacefill representation and the modelled parts of the SprE glycans shown as atomic spheres. Source data

**Extended Data Fig. 1:**
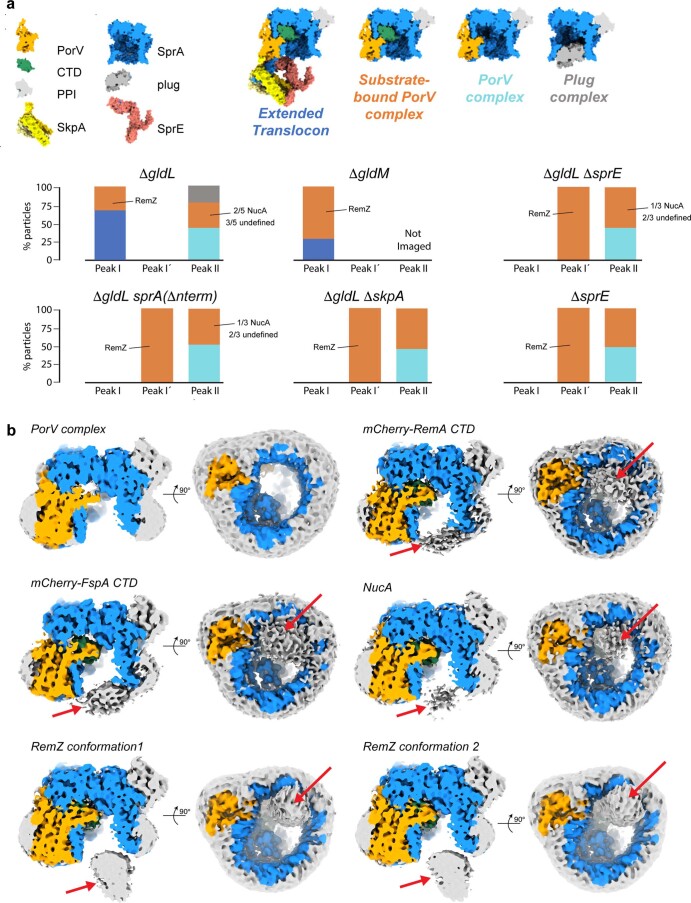
Comparison of substrate−containing translocon complexes. **a**, Graphical summary of the SprA complexes isolated from different genetic backgrounds. The bars in the graphs are coloured according to the structure cartoon key. The complexes and their relative abundances were determined by cryoEM analysis of the proteins in the indicated size−exclusions chromatography peaks. The identities of bound substrate molecule(s) are given where these can be assigned from the structural data. These substrate assignments are consistent with proteomics data (Extended Data Fig. 3a, Supplementary Data 1). RemZ (Fjoh_0803) is a homologue of the gliding adhesin RemA. NucA (Fjoh_4723) is a predicted nuclease. **b**, CryoEM volumes for substrate-containing translocon complexes isolated by *in vivo* trapping or *in vitro* reconstitution. The substrate-free PorV complex is shown for comparison. In each case the complex is shown in a cut-through of the protein viewed from within the plane of the membrane (left) and viewed from the periplasm (right). PorV, gold; SprA, blue; substrate CTD, green; PPI, detergent micelle, and unmodelled substrate regions, grey. Disordered substrate densities emerging from the SprA barrel are indicated with red arrows. The RemZ structures are those determined from the peak I′ fraction prepared from a Δ*gldL*Δ*sprE* background.

**Extended Data Fig. 2:**
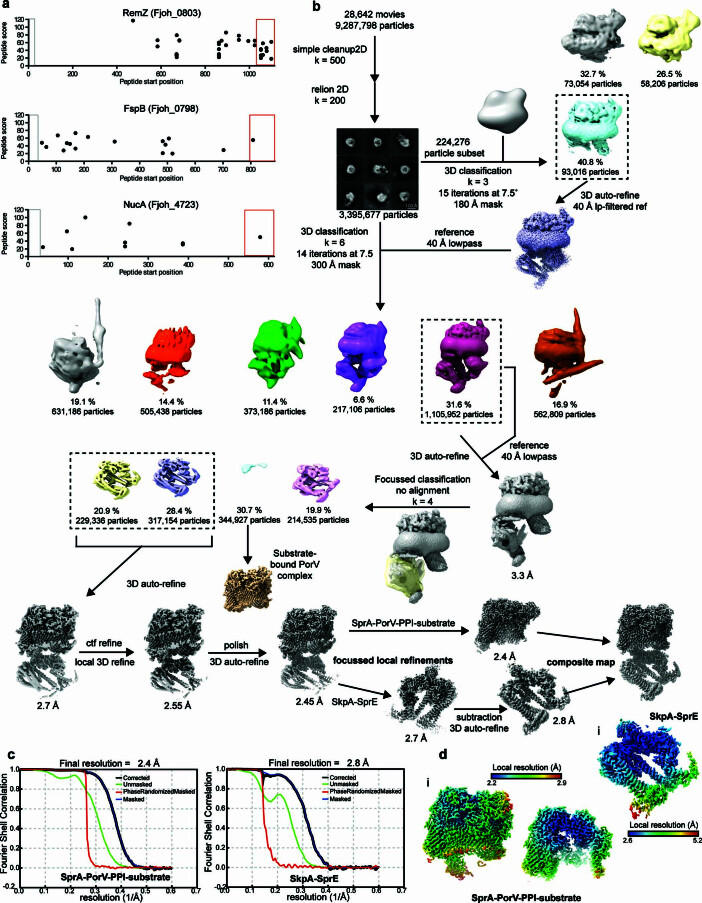
Proteomics characterization of the substrates present in SprA complexes purified from a Δ*gldL* backround and cryoEM workflow for the Extended Translocon structure. **a**, Peptide mass spectrometry of the substrate proteins most frequently associated with the SprA complexes isolated from a Δ*gldL* backround. The RemZ peptides are from direct sequencing of the ~110 kDa band of Peak I in Fig. 1b. The lack of coverage in the first part of the protein may reflect the paucity of trypsin sites in this region. The FspB and NucA peptides are from whole sample proteomics of Peak II in Fig. 1b. The peptide numbering is from the N terminus of the native precursor sequence. Regions of the proteins corresponding to the signal sequence are indicated by grey outline boxes and to the T9SS CTDs by red outline boxes. **b**, Image processing workflow for peak I of the SprA complexes purified from a Δ*gldL* background. **c**, Gold-standard Fourier Shell Correlation (FSC) plots calculated in Relion for the two focussed local refinements used to construct the composite Extended Translocon volume. **d**, The two focused local refinement volumes colored by local resolution estimates calculated in Relion. (i) The SprA-PorV-PPI-substrate volume is shown as both an intact (Left) and clipped (Right) surface. (ii) The SkpA-SprE volume.

**Extended Data Fig. 3:**
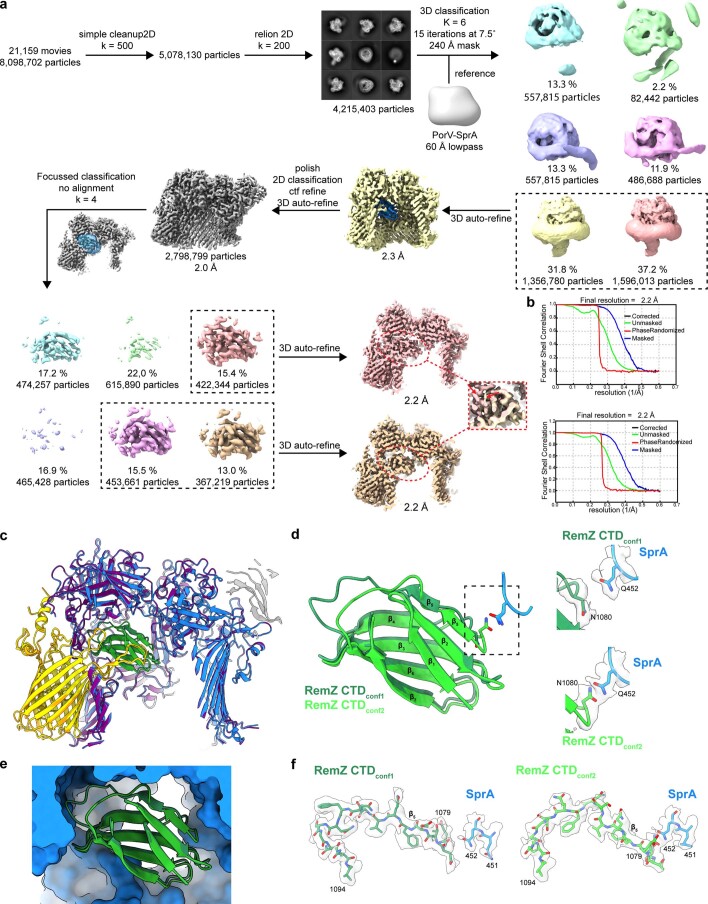
CryoEM analysis resolves two comformers of the RemZ CTD bound to the T9SS translocon. **a**, Image processing workflow for the Δ*gldL*Δ*sprE* peak I′ sample depicting separation of the two conformers of the RemZ CTD. **b**, Gold standard Fourier Shell Correlation plots calculated in Relion for the two conformer volumes. **c**, Slab view in cartoon representation through the models for the full RemZ-bound translocon complex with both conformers of the CTD present. Proteins are colored by conformer (conformer 1/conformer 2): SprA, blue/purple; PorV, yellow/orange; RemZ CTD, dark green/light green; PPI, grey/grey). **d**, Comparison of the interactions between the two RemZ CTD conformers and SprA Q452. Experimental densities are shown to the right for the region indicated by the dashed box. **e**, Closeup of the bound CTD showing both conformations in cartoon representation (dark green/light green) and with SprA shown in a surface representation (blue) with the front face removed. **f**, Density and model for the strand of RemZ that is remodelled between the two conformers.

**Extended Data Fig. 4:**
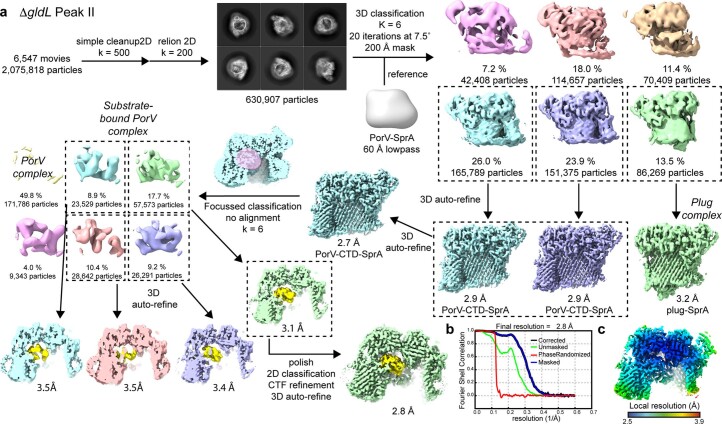
Workflow for the translocon complexes in peak II of the Δ*gldL* SprA purification. **a**, Image processing workflow for the SprA complexes in peak II of the Δ*gldL* SprA purification. **b**, Gold-standard Fourier Shell Correlation (FSC) plots calculated in Relion for the NucA-translocon complex structure determined from peak II of the Δ*gldL* SprA purification. **c**, The refinement volume of the NucA-translocon complex shown as a clipped surface and colored by local resolution estimates calculated in Relion.

To investigate the mechanism of protein transport by the T9SS, we sought to trap and characterize substrate-bound states of the translocon complex. One scenario in which we envisaged that substrate trapping might occur was if power input to the T9SS was blocked through disruption of the GldLM motor complex. To test this possibility, we carried out biochemical and cryogenic electron microscopy (cryo-EM) analyses of native *F. johnsoniae* SprA complexes isolated from a Δ*gldL* background. We found that around half of the recovered translocons contain a bound substrate protein (Fig. 1a,b, Extended Data Figs. 1a, 2, 3, 4a–c, and Supplementary Table 1 and Data 1). Some of these stalled complexes are larger than the previously isolated T9SS translocon (peak I in Fig. 1a) and contain additional protein components not seen in the earlier translocon preparations (Fig. 1b). We term these new complexes the Extended Translocon.

The core of the Extended Translocon corresponds to the previously characterized PorV complex that is composed of SprA, PorV and PPI^23^. However, this core is elaborated by additional components identified as the proteins SprE and Fjoh_1689 (Fig. 1b–d). SprE is a tetratricopeptide repeat (TPR)-containing lipoprotein^24,25^ and is equivalent to the *Porphyromonas gingivalis* T9SS component PorW^26^. Fjoh_1689 is a homologue of the *Escherichia coli* periplasmic chaperone Skp, hence we have chosen to call it ‘SkpA’. The analogous *P. gingivalis* protein (PGN_0300) has been implicated in Type 9 transport^27^.

SprE together with a SkpA trimer forms a dish-shaped periplasmic extension of the core translocon (Fig. 1c,d and Supplementary Video 1). The dish is attached perpendicular to the rim of the SprA cavity near the site of PorV incorporation, with the concave side of the dish facing the central axis of the cavity.

### Substrate binding to the T9SS translocon

**Fig. 2:**
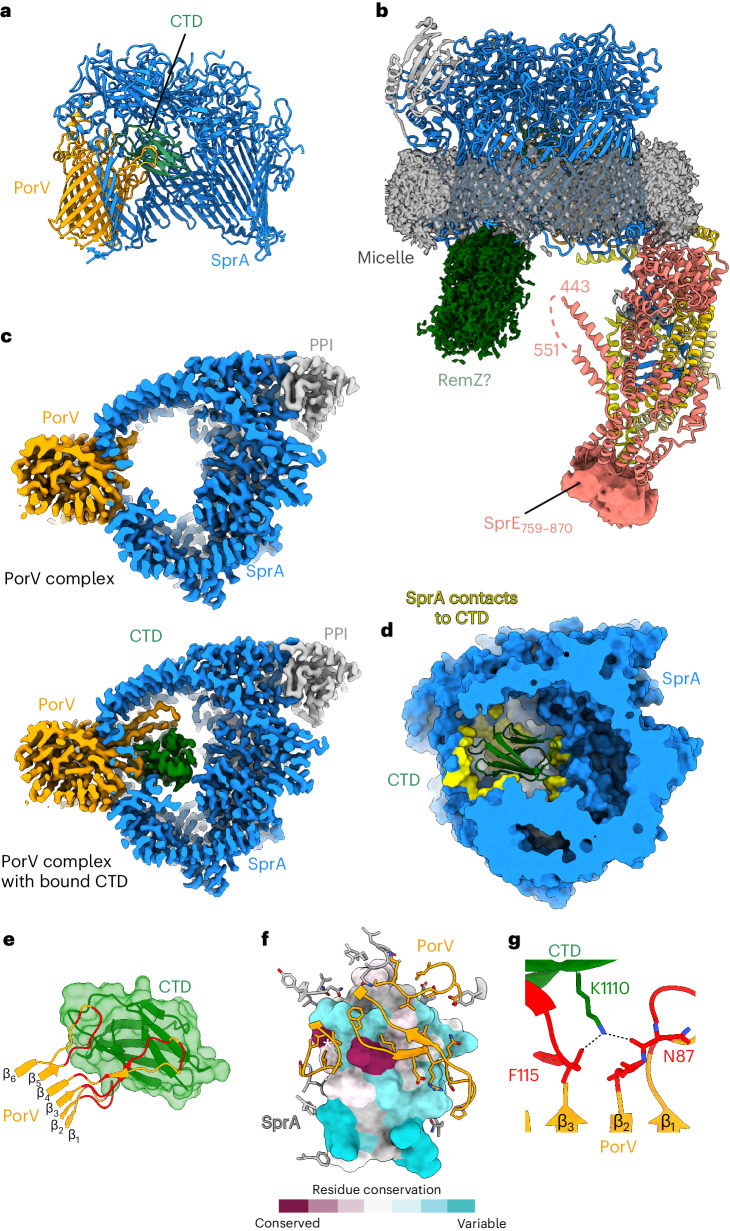
Substrate binding to the Extended Translocon. For clarity, only the core subunits of the Extended Translocon are shown in panels **a**, **c** and **d**. **a**, Cartoon representation with the front of the translocon cut away and the CTD shown as a green cartoon. **b**, The unmodelled EM volume overlaid on a cartoon representation of the Extended Translocon highlighting the detergent belt at the presumed location of the OM (dark grey volume), the unmodelled C-terminal domain of SprE (salmon volume) and the disordered density putatively identified as a portion of the trapped RemZ substrate (dark green volume). **c**, Equivalent slices through the cryo-EM volumes for the empty PorV complex (EMD-0133) (top) and the CTD-bound Extended Translocon (bottom) viewed from the periplasm. **d**, Horizontal slab view of SprA (blue) showing surfaces (yellow) in contact with the CTD (green ribbons). **e**, Interactions of the PorV loops with the CTD. **f**, The CTD surface in contact with the translocon is poorly conserved among type A CTDs except for a single Lys (position 1110 in RemZ) highlighted by a white *. Surface conservation was calculated using ChimeraX. **g**, Highly conserved RemZ Lys1110 forms a hydrogen bond with the main chain carbonyls of PorV Asn 87 and F115.

The substrate molecule trapped in the Extended Translocon was identified as the Type A substrate RemZ (Fjoh_0803), a homologue of the gliding adhesin RemA^28^. Only the CTD of this protein is well resolved in the structure. The CTD is bound within the SprA pore and has extensive interactions not only with the extracellular loops of PorV that protrude into the pore, as anticipated^23^, but also with SprA itself (Fig. 2). Nearly 40% of the surface of the CTD (~1,850 Å^2^) is buried on binding to the translocon, with ~850 Å^2^ buried in interactions with the PorV loops and ~1,000 Å^2^ buried in contacts with SprA. This very large buried surface area is nearly twice that of a typical antibody–antigen interface, implying a tight binding interaction. The substantial contribution of the SprA cavity to CTD binding by PorV suggests that the CTD will have a reduced affinity for PorV once it has left the translocon, and this could contribute to subsequent substrate release from PorV into the external environment. CTD binding occurs without major structural change to the translocon other than an ordering of the previously relatively disordered PorV loops as they wrap around the CTD (Fig. 2c,e).

**Extended Data Fig. 5:**
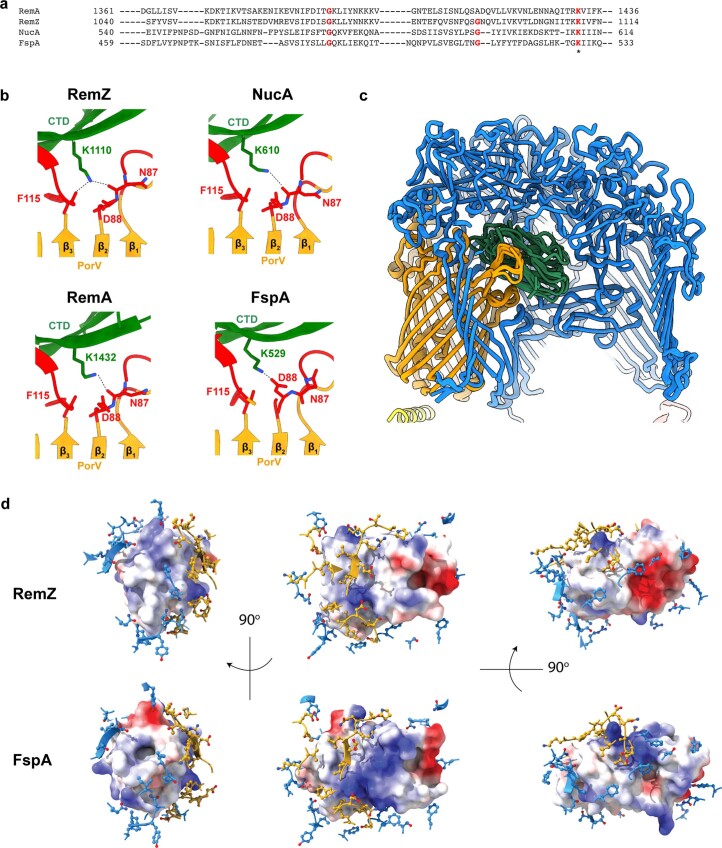
All CTDs studied bind in similar modes to the SprA/PorV complex. **a**, Structure-informed alignment of the sequences of the *F. johnsoniae* Type A CTDs that are structurally characterized in complex with the T9SS translocon in this work. The most conserved residues are highlighted in red. The highly conserved and functionally important lysine is indicated (*). **b**, The highly conserved lysine in Type A CTDs exhibits distinct interactions with PorV in different substrate-translocon complexes. **c**, The CTDs of different Type A substrates exhibit similar modes of binding to the translocon. Aligning the substrate-translocon structures determined in this work on SprA (blue) reveals only small variations in the positions of the substrate CTD (green) and PorV loops (orange). **d**, Nature of the CTD-translocon interaction illustrated for two substrate-translocon complexes. The electrostatic surface of the CTD domains is shown together with atom (side chain) and cartoon (backbone) representations of all SprA (blue) and PorV (orange) residues within 4 Å of that surface. The interactions are primarily with hydrophobic regions of the CTD surface.

Structural analysis of other stalled substrate-bound translocons from the motor-deleted strain shows that they bind Type A substrates in essentially the same manner as the Extended Translocon, and this is also true of further substrate–translocon complexes described below (Extended Data Figs. 1a and 5). Two slightly different conformers of the RemZ CTD that differ by a rethreading of one strand of the CTD can be resolved in the highest resolution map (Extended Data Fig. 3c–f), but this structural variation is unlikely to be biologically important because it does not impact on the binding interactions with PorV.

Our collection of Type A substrate–translocon structures allows us to assess how a single binding site in the translocon is able to recognize a range of substrate proteins bearing non-identical CTDs. Although Type A CTDs have conserved sequence motifs^1,5^, these residues do not mediate interactions with the translocon but instead specify the fold of the CTD (Fig. 2f). The only possible exception is an almost invariant and functionally important^5,16^ lysine residue close to the C terminus of the CTD (Fig. 2f and Extended Data Fig. 5a). In all our structures, the side chain of this residue forms a bonding contact with PorV. However, the interactions involved are not conserved (Fig. 2g and Extended Data Fig. 5b), suggesting that the primary role of this residue is elsewhere in the transport process. Our structures show that incoming CTDs are presented to an appropriately sized and shaped, but plastic, binding pocket. The regions of PorV and SprA that are involved in substrate recognition remodel to accommodate the different CTDs which they bind through predominantly hydrophobic interactions (Extended Data Fig. 5d).

No ordered density for the passenger domain of the substrate protein is resolved in any of the substrate-bound translocon structures. However, low thresholding reveals unassigned disordered density at the periplasmic rim of the SprA cavity that probably arises from these domains (Figs. 1c and 2b, Extended Data Fig. 1b and Supplementary Video 1). Thus, while the passenger domains do not interact in a tight or specific fashion with the SprA cavity, they are non-randomly positioned within the translocon.

### The periplasmic domain of the Extended Translocon

**Fig. 3:**
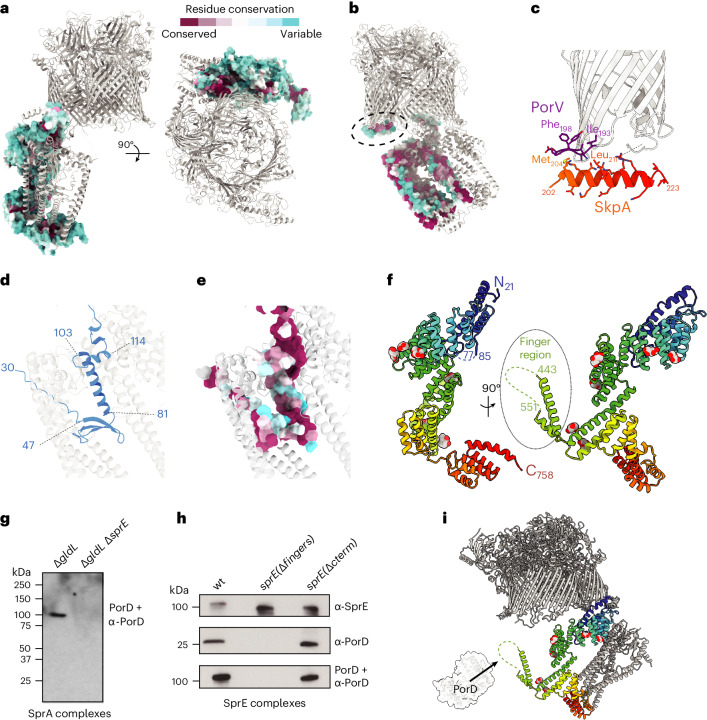
Structural features involved in the assembly of the Extended Translocon. **a**,**b**, The surfaces of SprE (**a**) and SkpA (**b**) that interact with other translocon components are highly conserved. Surface conservation was calculated using ConSurf^62^. **c**, Closeup in ribbon representation of the region highlighted by a dashed oval in **b** showing the interaction between PorV and the ordered C-terminal helix of one copy of SkpA. Residues involved in the contact are labelled. **d**,**e**, The N terminus of SprA (**d**, blue) folds within the core of the SkpA chamber. Residues critical for formation of this fold, or that interact with SkpA, are highly conserved as assessed with ConSurf^62^ (**e**). **f**, Cartoon representation of SprE in rainbow colouring from the N (blue) to C terminus (red). The modelled portions of the glycans are shown as atomic spheres. **g**,**h**, PorD is bound to the Finger Region of SprE. Twin-strep–SprA (**g**) or SprE–Twin-strep (**h**) complexes were affinity purified from the indicated backgrounds where *sprE(*Δ*fingers)* and *sprE(*Δ*cterm)* are deletions of the structurally unresolved regions of the SprE Finger Region and C terminus respectively. The complexes were immunoblotted for SprE–Twin-strep (α-SprE), or PorD (α-PorD) or far western blotted by incubation with PorD followed by immunodetection of PorD (PorD + α-PorD). Similar data were obtained from two independent preparations. **i**, Inferred location of PorD (represented by an AlphaFold2^63^ model) in the Extended Translocon complex. PorD interacts with the disordered portion of the SprE Finger Region (dashed line). Source data

The periplasmic domain of the Extended Translocon is tied to the translocon core primarily by interactions with the extended N terminus of SprA, a region of the protein that was not resolved in the PorV and Plug complex structures^23^. The SkpA trimer encapsulates the SprA N terminus, and there are additional interactions between the base of the SprA N terminus and the most N-terminal part of SprE (Figs. 1c,d and 2b). Further interactions between the dish and the core translocon are restricted to contacts between two N-terminal regions of SprE and the rim of the SprA cavity (Figs. 1d, 2b and 3a), and by an interaction between a helix in one copy of SkpA and the base of PorV (Figs. 1d and 3b,c). Despite the limited contacts with the rim of the SprA cavity, this network of interactions ties the periplasmic extension tightly to the core complex. Notably, the surfaces of SprE and SkpA that interact with other translocon components are highly conserved (Fig. 3a,b).

The trimeric SkpA component of the Extended Translocon has the same ‘jelly-fish’ structure as the canonical *E. coli* Skp homotrimer in which the N termini of the subunits interact to form a ‘body’ from which are hung three long α-helical ‘tentacles’^29^ (Figs. 1c, 2b and 3b). In the *E. coli* Skp complex the tentacles trap client proteins within the central cavity where they are held in a highly dynamic and unstructured state^30,31^. By contrast, the SkpA protein in the Extended Translocon is used to immobilize and structure an otherwise disordered polypeptide, namely the N terminus of SprA (Figs. 1d and 3d,e). The SprA residues involved in the formation of this N-terminal fold or in its interactions with SkpA are highly conserved (Fig. 3e).

**Extended Data Fig. 6:**
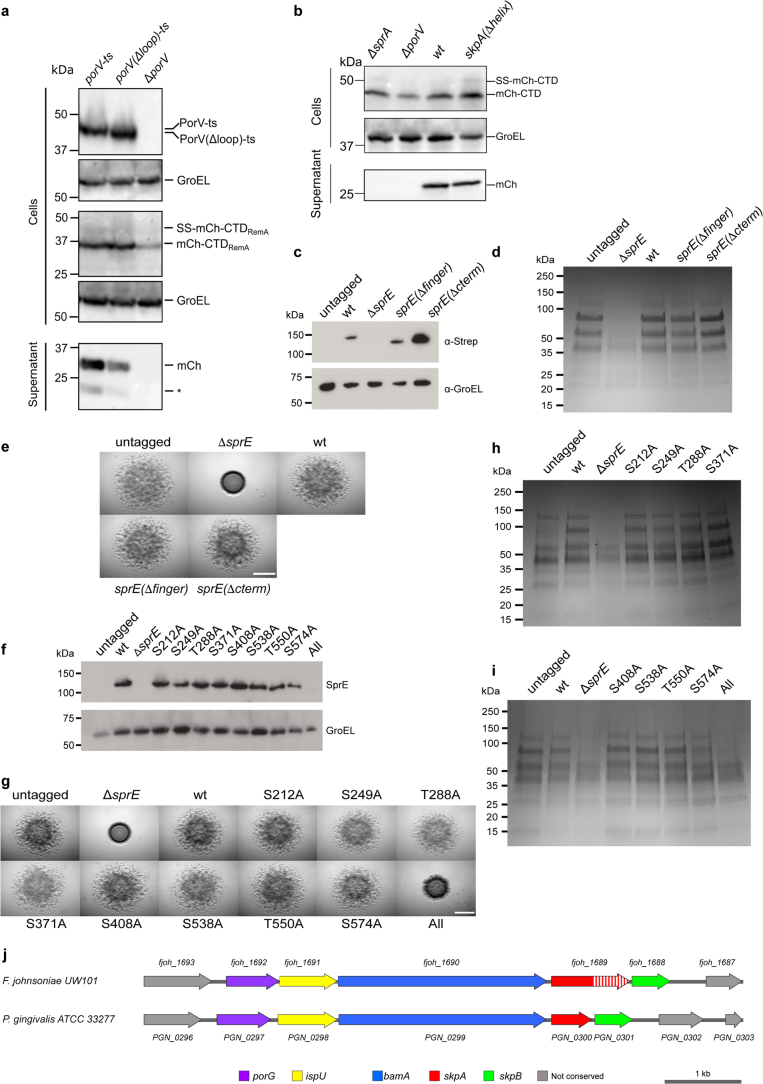
Characterization of SprE, SkpA, and PorV variants. **a, b**, Functional analysis of the contact between the C-terminus of SkpA and the periplasmic face of PorV assessed by (**a**) deletion of the PorV periplasmic loop (residues 193-199, allele *porV(*Δ*loop)*) or (**b**) deletion of the C-terminal SkpA helix (residues 203-218, allele *skpA(*Δ*helix)*). The PorV (**a**) or SkpA (**b**) proteins used in these experiments have C-terminal or N-terminal Twin-Strep epitope tags, respectively. T9SS export was assessed using a model substrate protein comprising a signal sequence (SS), mCherry (mCh), and the T9SS-targeting C-terminal domain of RemA (CTD). Cells and culture supernatants were separated and analysed by immunoblotting. The substrate protein was detected using mCherry antibodies and PorV-TwinStrep was detected by TwinStrep antibodies. PorV and mCherry in (**a**) were analysed from the same samples but on duplicate immunoblots, each with a GroEL loading control. Wt, wild type strain. *, mCh degradation product. **c-i**, Characterization of SprE variants. All *sprE* mutants were constructed in a *sprE*-*twinstrep* background (wt). A strain with untagged *sprE* (untagged) is included as a control. **c-e**, The Finger Region and C-terminal region of SprE are not required for T9SS function. Strains expressing SprE with either the Finger Region (residues E445 to T550) replaced with a (GS)_10_ peptide (*sprE(*Δ*finger)*) or with the C-terminal tail region removed (residues F759 to P870, *sprE(*Δ*cterm)*) were analysed. **f-i**, *O*-glycosylation is important for SprE stability. SprE variants with the indicated single amino acid substitutions of identified (structurally or [S538] biochemically^32^) or predicted (T550 from a previously identified Bacteroidota *O*-glycosylation motif^32,33^) glycosylated residues, or containing all eight substitutions (‘All’) were analyzed. **c, f**, Whole cell immunoblotting for SprE-TwinStrep variants. GroEL serves as a loading control. **d, h, i**, Secretome analysis of culture supernatants. The samples were separated by SDS-PAGE and stained with Coomassie Blue. **e, g**, Spreading (gliding) morphology of colonies on agar. Scale bar: 4 mm. **j**, The structure of the *skpA*-containing operon in *F. johnsoniae* and *P. gingivalis*. A sequence extension of *F. johnsoniae skpA* relative to *P. gingivalis skpA* is shown hatched. Attempts to delete *skpB* in *F. johnsoniae* and *P. gingivalis* have been unsuccessful (our observations and 27) suggesting that SkpB is an essential protein in these organisms. **a-i**, Similar results were observed in three biological repeats. Source data

The *F. johnsoniae* SkpA proteins are extended at the C terminus relative to *E. coli* Skp. Although these extensions are mainly unresolved in the structure, they provide the SkpA helix that contacts PorV (Figs. 1d and 3b,c). To investigate whether this interaction is important for T9SS function we constructed strains disrupting each side of the contact. However, a decrease in the rate of Type 9 export was only observed in the mutant targeting the PorV side of the interaction (Extended Data Fig. 6a,b), suggesting that the PorV–SkpA helix contact is not essential for T9SS function under our assay conditions.

SprE forms an extended TPR-containing scaffold with 13 TPR units in the structurally well-defined portion of the molecule (Fig. 3f). The ninth repeat is interrupted by a 160-amino acid insertion which we term the Finger Region. This comprises ~100 unresolved amino acids sandwiched between two α-helical ‘fingers’ which point across the SprA pore towards the unresolved substrate density (Fig. 2b). The final 113 amino acids of SprE are not well ordered in the EM map (Fig. 2b). Although SprE is essential for Type 9 secretion^24,25^, replacement of the central disordered portion of the Finger Region with a stuffer peptide, or truncation of SprE to remove the poorly resolved C-terminal region, had no detectable effect on T9SS function (Extended Data Fig. 6c–e). Thus, the structurally unresolved portions of SprE are not essential for Type 9 protein transport. SprE is glycosylated (Figs. 1d and 3f, and Extended Data Fig. 6f–i)^32,33^.

Proteomics analysis of the pool of SprA complexes containing the Extended Translocon identifies a structurally unassigned protein PorD (Fjoh_3466) (Fig. 1b). This protein has a phylogenetic distribution that strongly correlates with the presence of a T9SS^26,34^ and was recently reported to influence processing of T9SS substrate proteins in *P. gingivalis*^35^. Far western blotting shows that PorD is bound to SprE (Fig. 3g). Further analysis shows that PorD binds within the disordered part of the SprE Finger Region (Fig. 3h), explaining why PorD is not visible in the EM structure (Fig. 3i).

### Functional importance of the Extended Translocon components

**Fig. 4:**
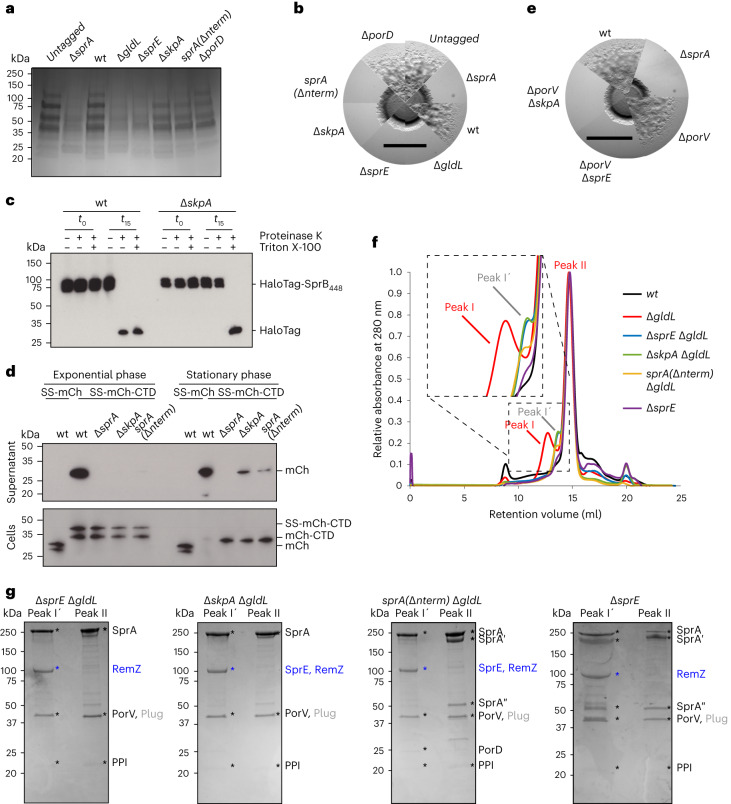
Functional importance of the Extended Translocon components. **a**, Secretome analysis of culture supernatants. The samples were separated by SDS–PAGE and stained with Coomassie blue. **b**,**e**, Spreading (gliding) morphology on agar of Extended Translocon component mutants in the presence (**b**) or absence (**e**) of *porV*. Scale bars, 4 mm. **c**, Cell surface exposure of the adhesin SprB assessed by protease protection. Strains expressing a fusion between HaloTag and the final 448 amino acids of SprB (HaloTag-SprB_448_), and containing a Δ*porV* mutation to reduce endogenous proteolysis, were incubated with Proteinase K and the detergent Triton X-100 as indicated. Reactions were stopped immediately (*t*_0_) or after 15 min (*t*_15_) and analysed by immunoblotting with anti-Halotag antibodies. **d**, Analysis of the secretion of a model substrate protein comprising a fusion between a signal sequence (SS), mCherry (mCh) and the T9SS-targeting C-terminal domain of RemA (CTD). SS-mCh is a control fusion protein lacking a CTD. Cells were grown to exponential or stationary phase as indicated, separated into cell and supernatant fractions and analysed by anti-mCherry immunoblotting. The successively processed forms of the fusion proteins are indicated to the right of the blots. **f**,**g**, Effects of removing Extended Translocon components on SprA complex composition. **f**, Size exclusion chromatography profiles of Twin-strep–SprA complexes purified by Streptactin-affinity chromatography from the indicated strains (wt and Δ*gldL* background profiles are from Fig. 1a). The inset shows a magnified view of Peak I and Peak I′. **g**, Coomassie-stained SDS–PAGE gels of the peak fractions from **f**. The composition of the ~100 kDa band (blue labels) was determined by peptide fingerprinting. The identities of other bands were assigned by analogy to the preparations in Fig. 2b and supported by whole-sample peptide fingerprinting (Supplementary Data 1). SprAʹ and SprAʺ indicate fragments of SprA that arise from proteolytic clipping^23^. Similar data were obtained from two independent preparations. **a**–**d**, Similar data were obtained from three biological repeats. **a**,**b**,**e**–**g**, A *twin-strep–sprA* background was used (wt) except where indicated (untagged). Source data

We individually deleted the Extended Translocon-specific proteins and assessed the effects on T9SS function through an analysis of the secreted proteome (Fig. 4a) and by examining gliding motility on agar which depends on the T9SS-secreted adhesin SprB (Fig. 4b). In agreement with previous reports^24,25^, T9SS function was abolished by removal of SprE. By contrast, the absence of PorD had no detectable effect on T9SS activity. The phenotypic effects of the removal of SkpA on T9SS function were more complex, with gliding on agar completely abolished but the secreted proteome only partially depleted. Further investigation showed that SkpA was essential for the export of the surface adhesin SprB, consistent with the gliding defect of these cells (Fig. 4c). Similarly, exponentially growing cells of the Δ*skpA* mutant failed to secrete a model Type A CTD-containing substrate protein (Fig. 4d). However, if growth of the mutant cells was extended to late stationary phase to match the conditions used for the secretome analysis, then trace export of the test protein was detected (Fig. 4d). Thus, Type 9 export depends on SkpA but this requirement is not absolute at late growth stages.

If binding of SkpA to the Extended Translocon is blocked by removing the N terminus of SprA, then Type 9 transport is affected to the same extent as removing SkpA (Fig. 4a,b,d). This shows that the presence of SkpA within the Extended Translocon is important for Type 9 transport irrespective of any chaperone functions that SkpA might be providing.

Although the Extended Translocon contains the Type A carrier protein PorV, removing SprE or SkpA not only prevents the export of Type A substrates but also the Type B substrate SprB which uses an alternative carrier protein (Fig. 4b,c). Thus, the roles of SprE and SkpA in Type 9 secretion are not restricted to PorV-containing translocon complexes. We can exclude the possibility that the loss of these proteins indirectly prevents SprB export through blocking the SprA channels with stalled PorV-bound substrate proteins, because removing PorV fails to restore SprB-dependent gliding motility to Δ*sprE* and Δ*skpA* mutants (Fig. 4e).

**Extended Data Fig. 7:**
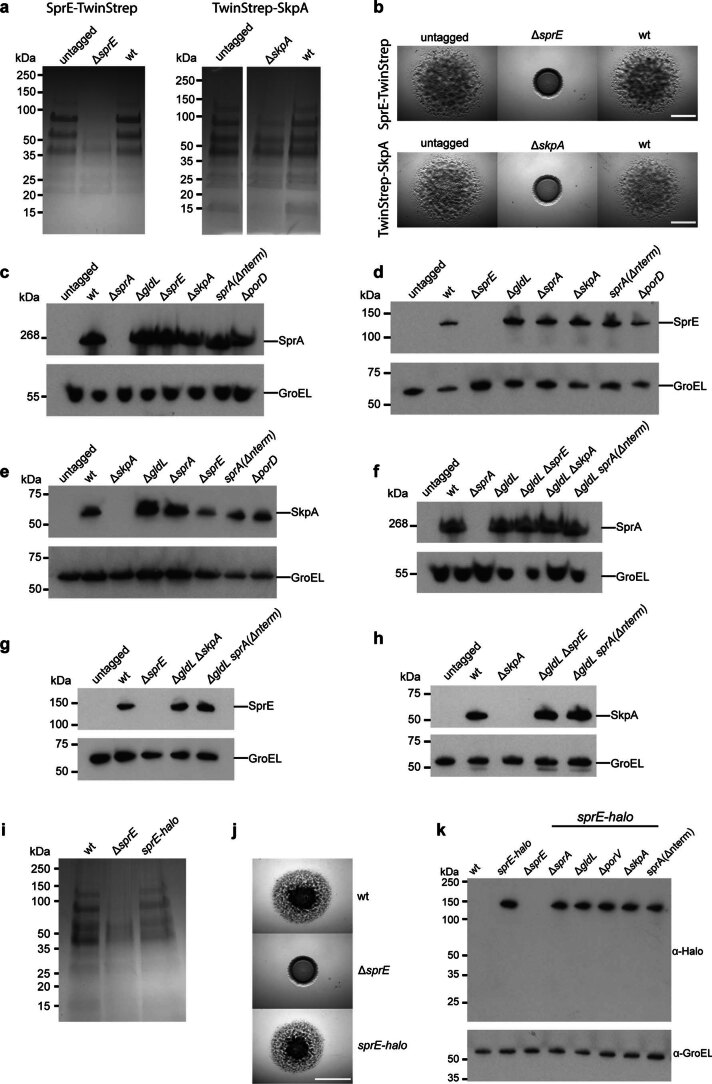
Analysis of the function and expression levels of fusions to Extended Translocon components. **a-h**, The protein of interest in each panel is epitope tagged with a TwinStrep peptide. The strain with the tagged protein was used as the wild type (wt) background for the mutant strains. A strain in which the protein of interest has not been epitope tagged is labelled ‘untagged’. **i-k**, Test strains have a HaloTag coding sequence fused to the end of the chromosomal *sprE* gene (*sprE-halo* allele). wt, wild type. **a, b, i, k**, TwinStrep epitope tagging of SprE or SkpA or HaloTag fusion to SprE does not affect Type 9 secretion or gliding function. **a, i**, Secretome analysis of culture supernatants. The samples were separated by SDS-PAGE and stained with Coomassie Blue. **b, k**, Spreading (gliding) morphology of colonies on agar. Scale bar: 4 mm. **c-h**, Analysis of the effects of removal of Extended Translocon components on the levels of other Extended Translocon components. Whole cell immunoblotting with anti-Strep antibodies for the presence of TwinStrep-tagged SprA (**c,f**), SprE (**d,g**), and SkpA (**e,h**) in the indicated genetic backgrounds. Immunoblotting with anti-GroEL antibodies serves as a loading control. **k**, Whole cell immunoblotting of the indicated strains with Halotag (α-Halo) and GroEL (α-GroEL) antibodies. GroEL serves as a loading control. Similar data were obtained for two (blots) or three (other analyses) biological repeats. Source data

Purification of SprA complexes from strains deleted for both the motor complex and individual Extended Translocon components shows that there is interdependence between SprE and SkpA in their stability of binding to SprA but that their loss does not prevent substrate trapping on the translocon (Fig. 4f,g and Extended Data Fig. 7).

### Factors affecting substrate binding by the Type 9 translocon

**Extended Data Fig. 8:**
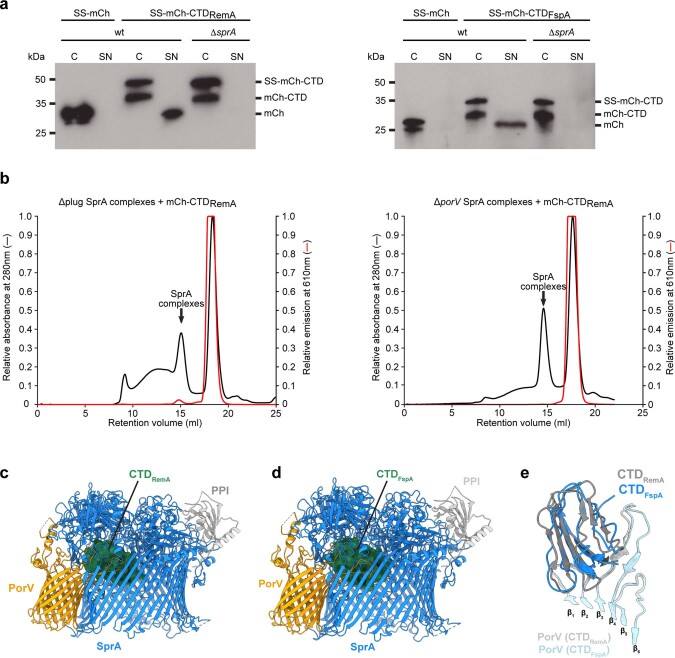
*In vitro* reconstitution of substrate protein binding to the T9SS translocon. **a**, Model substrate proteins are transported by the T9SS. Analysis of the export of model substrate proteins from wild-type *F. johnsoniae* cells (wt) or a strain lacking the T9SS translocon (Δ*sprA*). The substrate proteins consist of a tripartite fusion between a signal sequence (SS), mCherry (mCh), and the T9SS-targeting C-terminal domain of either RemA (CTD_RemA_) or FspA (CTD_FspA_). The two left hand lanes show experiments with a control fusion protein lacking a CTD (SS-mCh). Cells were grown in MM medium for 4 h, separated into cell (C) and supernatant (SN) fractions, and analyzed by anti-mCherry immunoblotting. The successively processed forms of the fusion proteins are indicated. Removal of the signal sequence marks transport across the inner membrane by the Sec apparatus and removal of the CTD marks transport across the outer membrane by the T9SS. Similar data were obtained for at least two biological repeats. **b**, *In vitro* reconstitution of RemA CTD binding to SprA complexes. Size exclusion chromatography profiles of affinity-purified TwinStrep-SprA complexes from wild type (wt, left panel) and Δ*porV* mutant (right panel) strains following incubation with a mCherry-CTD_RemA_ fusion protein. The elution profile of the total protein content is indicated in black (relative absorbance at 280 nm), and the elution profile of mCherry-CTD_RemA_ is indicated in red (fluorescence emission at 610 nm following excitation at 587 nm). The peak containing TwinStrep-SprA complexes is indicated. **c,d** Cartoon representation of the (**c**) mCherry-CTD_RemA_ and (**d**) mCherry-CTD_FspA_ complexes. The bound CTD is shown in surface representation (dark green) within the SprA barrel (blue). **e**, Overlay of cartoon representations of the CTDs and PorV loops from the translocon complexes containing the RemA and FspA CTDs. Source data

**Extended Data Fig. 9:**
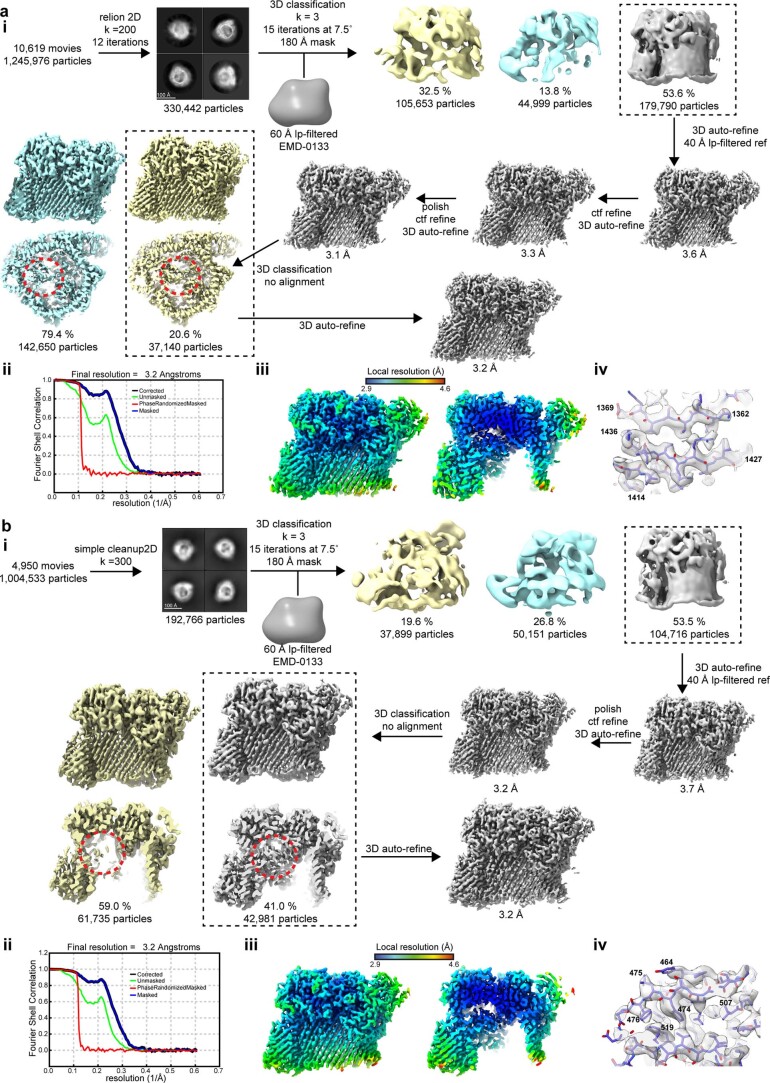
Cryo-EM processing workflows for *in vitro* reconstituted model substrate-translocon complexes. **a, b**, (i) Image processing workflow. (ii) Gold-standard Fourier Shell Correlation (FSC) plot used for global resolution estimation as determined with Relion. (iii) Volume colored by local resolution estimate as determined within Relion. (iv) Closeup of model and volume for the bound CTD domain within the SprA barrel. **a**, mCherry-CTD_RemA_-translocon complex. **b**, mCherry-CTD_FspA_-translocon complex.

Our data show that substrate proteins accumulate on the translocon when the T9SS energy source is removed either alone or in combination with the loss of SprE or SkpA (Figs. 1 and 4g, and Extended Data Fig. 1). This implies that substrate loading onto the translocon does not require energization, or SprE, or SkpA. To directly test this conclusion, we asked whether model Type A substrate proteins are able to bind to purified SprE-free and SkpA-free PorV complexes in vitro in the absence of energetic input. Substrate binding to the translocon was indeed observed, and structural analysis confirmed that the in vitro-reconstituted complexes bind substrate proteins in the same way as the in vivo-trapped complexes (Extended Data Figs. 8 and 9).

We infer that energy is required to extract substrates from the translocon because substrates are trapped on the translocon when the transport motor is absent. We also see substrate trapping if we remove the SprE component of the Extended Translocon (strain Δ*sprE* in Fig. 4f,g, Extended Data Figs. 1a, 4e,k and 7c–e, and Supplementary Table 1 and Data 1). This indicates that the dish components SprE and/or SkpA (which is also lost from the translocon in this background) are necessary for substrate release even when the motor complex is intact.

### The physiologically relevant form of the Type 9 translocon

SprE and SkpA are essential for Type 9 transport. Nevertheless, complexes containing these proteins comprise only a small proportion of the translocons isolated from a Δ*gldL* background and are not present in the translocon complexes purified from the wild-type strain (Fig. 1b). This contradiction could be explained if SprE and SkpA dynamically associate with SprA during the transport cycle and are partially trapped in this assembled state when the transport motor is removed. Alternatively, SprE and SkpA might be constitutively associated with the translocon but their association with SprA is only stable to purification in the special case that the translocon contains trapped RemZ substrate. To distinguish between these two possibilities, we used single-molecule tracking to assess whether SprE is continuously or dynamically associated with SprA in living cells. Any SprE molecules that are bound to SprA will be static because SprA is immobile in the OM^23^. Conversely, SprE molecules that are not associated with SprA will be able to diffuse along the surface of the OM to which they are attached through their lipid anchor.

**Fig. 5:**
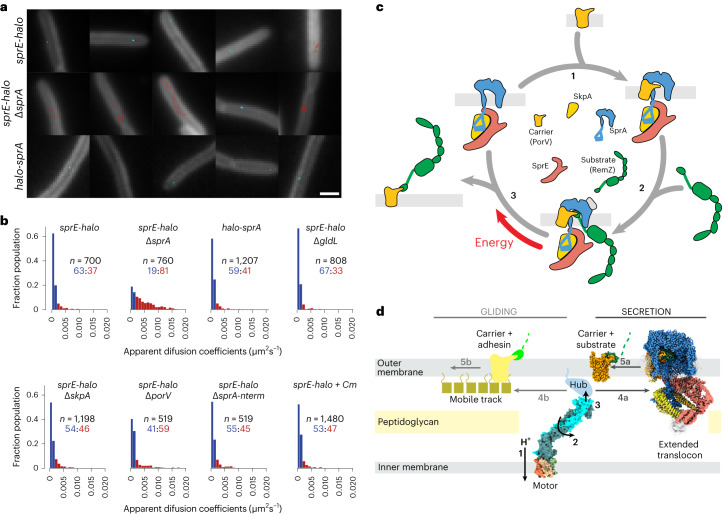
Protein–protein interactions during protein export by the T9SS translocon. **a**,**b**, Single-molecule tracking of fluorophore-labelled HaloTag-SprA or SprE-HaloTag in live *F. johnsoniae*. Images were acquired by stroboscopy with a 50 ms frame rate and a one-frame-on-six-frames-off cycle. **a**, Representative trajectories in the indicated strains. The trajectories link the single-molecule localizations in successive frames. Trajectories are coloured as ‘immobile’ (cyan) or ‘mobile’ (red) according to the classification in **b**. Trajectories are 8.45 s in duration (25 frames). Scale bar, 1 μm. See also Supplementary Video 2. **b**, Distributions of the apparent diffusion coefficients (D*) of individual tracked molecules in the indicated backgrounds. Molecules with D* ≤ 0.0017 μm^2^ s^−1^ are classed as ‘immobile’ (blue) and molecules with D* above this value classed as ‘mobile’ (red). The number of tracks analysed (*n*) and the percentages of immobile and mobile molecules (blue:red) are given above each distribution. The protein synthesis inhibitor chloramphenicol was added to ‘+Cm’ cells. **c**, Updated model of the T9SS translocon mechanism for Type A substrates based on the results of this study. Substrates with a Type B CTD are anticipated to utilize an analogous cycle employing an alternative carrier protein. The Extended Translocon, shown here as composed of SprA, SprE and SkpA, is the physiologically relevant translocation complex. (1) PorV docks onto the lateral opening of SprA. (2) The CTD of a substrate protein binds to the loops of PorV that protrude into the SprA pore, assisted by interactions between the CTD and the SprA cap. (3) Energetic input forces PorV away from the translocon, pulling the PorV-bound substrate molecule through the lateral opening into the extracellular environment. **d**, Schematic of the energy chain that drives both T9SS protein transport and gliding motility. A motor complex transduces the energy of protons flowing down their electrochemical gradient (1) into rotary mechanical energy (2) which it transfers through a periplasm-spanning arm to a Hub complex at the OM (3). The Hub complex distributes this mechanical energy both to the Extended Translocon (4a) to extract carrier protein–substrate complexes (5a) and to a mobile track (4b) that propels carrier protein-anchored surface adhesins (5b).

To enable fluorescent labelling of SprE, we fused a HaloTag coding sequence to the chromosomal *sprE* gene. The resultant fusion is fully functional and stable (Extended Data Fig. 7i–k). In a wild-type background the SprE-HaloTag fusion proteins were predominantly immobile and their overall mobility distribution closely matched that of a HaloTag-SprA fusion (Fig. 5a,b and Supplementary Video 2). These observations are consistent with the SprE molecules being associated with SprA translocons. If SprA was removed from the cells, the SprE-HaloTag fusion became considerably more mobile (Fig. 5a,b and Supplementary Video 2). This observation confirms that the immobilization of SprE observed in wild-type cells is due to interactions with SprA. Taken together these data indicate that at steady state SprE is predominantly bound to SprA. To assess whether SprE, nevertheless, transiently dissociates from SprA during the transport cycle, we measured the mobility of SprE-HaloTag in cells either blocked for substrate release from the translocon by removing the motor protein (a Δ*gldL* background), or blocked for substrate uptake (a Δ*porV* background), or treated with the protein synthesis inhibitor chloramphenicol to exhaust substrate availability. In all cases, the mobility distributions were similar to those of SprA-associated SprE rather than that of uncomplexed SprE (Fig. 5b). Thus, our data indicate that SprE is associated with SprA in cells throughout the transport process and that the Extended Translocon is the physiologically relevant form of the T9SS translocon.

SprE-HaloTag also remained predominantly immobile when SkpA was removed from the Extended Translocon (Δ*skpA* and *sprA(*Δ*nterm)* backgrounds, Fig. 5b), indicating that the interactions between SprE and SprA are sufficient to maintain their binary complex in the absence of SkpA in cellulo.

## Discussion

We show here that the T9SS translocon complex identified in earlier work^23^ is part of a much larger Extended Translocon structure that also contains the periplasmic proteins SprE, PorD and a SkpA trimer. This Extended Translocon may, in turn, be part of an even larger structure since it has recently been reported that the *P. gingivalis* SprE analogue PorW interacts with the Hub complex of the T9SS, which itself appears to be a constituent of a yet larger translocation site supercomplex^18,35^. Curiously, however, the C-terminal region of *P. gingivalis* PorW that was identified as interacting with the Hub in that study^35^ appears not to be required for Type 9 transport^36^ and the equivalent region in *F. johnsoniae* SprE is neither conserved nor functionally essential (this work). A recent proteomics study that identified interactions between the *P. gingivalis* proteins PorW, PorD and the SprA homologue Sov is consistent with the composition of the Extended Translocon structure reported here but infers a different PorD binding site on SprE^35^.

A surprising feature of the Extended Translocon is that it incorporates a homologue (SkpA) of the paradigmatic *E. coli* periplasmic chaperone Skp^37^. The presence of this protein in the translocon complex is unlikely to be an adventitious interaction because removal of the SkpA binding site on SprA abolishes Type 9 transport. Furthermore, SkpA has conserved packing contacts with the other Extended Translocon components, and the previously disordered N terminus of SprA becomes structured within the SkpA substrate cavity in contrast to the dynamic interactions observed with typical Skp clients^30^ (Fig. 3b–e). The only currently established roles for Skp are in the biogenesis of OM proteins^37–40^. However, the function of SkpA in the Extended Translocon is not OM protein biogenesis because the SprA and PorV barrel components of the translocon are still successfully inserted in the membrane when SkpA is absent (Fig. 4f,g, and Extended Data Figs. 1a and 7c). Instead, the Skp scaffold has been repurposed as a structural element within the translocon where it plays a critical role in determining the conformation and location of SprE. In addition to its role in the T9SS, SkpA is likely to have further functions in the cell because the operon encoding SkpA is conserved even in those Bacteroidota species that do not have a T9SS. The *skpA*-containing operon also codes for a second Skp homologue (Extended Data Fig. 6j). This protein is unable to substitute for SkpA in supporting Type 9 transport but is an essential protein (Extended Data Fig. 6j and ref. ^27^) in contrast to the canonical *E. coli* Skp protein^41^ or SkpA. Taken together, these observations suggest that the biology of Skp proteins is more complex than has been appreciated from studies in *E. coli* and that Skp proteins have multiple functional roles that remain to be fully characterized.

Structural analysis of the translocation intermediates trapped in this work shows that substrate binding occurs within the translocon cavity. This binding is mediated by the substrate CTD which interacts both with the walls of the cavity and with the extracellular loops of the PorV carrier protein that protrude into the cavity through the lateral opening. This mode of binding implies that substrate proteins exit the pore of the translocon CTD-first through the lateral opening in complex with PorV, as previously anticipated^23^.

Type 9 protein transport is driven by the PMF across the IM^16^ but the role that this energetic input performs in the transport mechanism has not been established. We found that we could trap substrate proteins on the translocon by removing the transport motor. The most parsimonious interpretation of this observation is that energy is needed for substrate extraction from the translocon but not for substrate loading. Thus, substrate release from the translocon is the energy-requiring step in Type 9 protein transport. This conclusion contrasts with the recent suggestion that the role of the motor complexes is to drive substrates onto the translocon^3^.

Taken together, our data show that during Type 9 transport, the substrate is recognized directly by its cognate carrier protein at the translocon and an energetic input is then used to extract this carrier protein–substrate complex from the translocon to complete the movement of the substrate protein across the OM (Fig. 5c).

The energetic input to the translocon is delivered through the energy chain (Fig. 5d). Logically this process should utilize the same mechanism that the energy chain uses to power gliding motility (Fig. 5d). During gliding motility the energy chain drives the movement of mobile tracks on which the gliding adhesins ride^16,42–44^ (Fig. 5d). The adhesins are Type B T9SS substrates which are linked to the gliding tracks by the carrier proteins that were used to transport them to the cell surface^13,14^. Thus, during gliding, the function of the energy chain is to move a carrier protein-propelling track. By analogy, we propose that in Type 9 transport the energy chain causes a moving track to attach to substrate-loaded carrier proteins to pull them away from the translocon. Notably, in the case of gliding adhesins, our proposal allows the same mechanistic event to accomplish both adhesin transport and loading of the adhesin onto the moving track network. We infer that the moving track for Type 9 transport is the Hub complex because the GldK/PorK component of the Hub is structurally related to the GldJ protein that forms the gliding tracks^44,45^. In *P. gingivalis*, the Hub complex has been shown to be a large ring structure^17,18^ and so may rotate to repetitively displace CTD-binding carrier proteins from the translocon. Further experimental work will be needed to validate this speculative model.

## Methods

### Bacterial strains and growth conditions

All strains and plasmids used in this work are listed in Supplementary Table 2. *F. johnsoniae* was routinely grown aerobically in Casitone yeast extract (CYE) medium^46^ at 30 °C with shaking. For some physiological studies, the cells were cultured in motility medium (MM)^47^ or PY2 medium^48^ as indicated below.

### Genetic constructs

Plasmids were constructed by Gibson cloning^49^ or Q5 site-directed mutagenesis (New England Biolabs) using the primers and target DNA in Supplementary Table 3. Suicide and expression plasmids were introduced into the appropriate *F. johnsoniae* background strain by biparental mating, using *E. coli* S17-1 (ref. ^50^) as donor strain as previously described^46^. Chromosomal modifications were introduced using the suicide vector pYT313 harbouring the counter-selectable *sacB* gene as previously described^16^. All plasmid constructs and chromosomal modifications were confirmed by sequencing.

### Purification of SprA and SprE complexes

To purify complexes containing Twin-strep-tagged SprA, the relevant strain was cultured for 22 h in 12 l of CYE medium using 500 ml culture volume in 2 l flasks. Cells were collected by centrifugation at 12,000 *g* for 30 min and stored at −20 °C until further use. All purification steps were carried out at 4 °C. Cell pellets were resuspended in buffer W (100 mM Tris-HCl, pH 8.0, 150 mM NaCl, 1 mM EDTA) containing 30 μg ml^−1^ DNase I, 400 μg ml^−1^ lysozyme and 1 mM phenylmethylsulfonyl fluoride (PMSF) at a ratio of 5 ml of buffer to 1 g of cell pellet. Cells were incubated on ice for 30 min with constant stirring before being lysed by three passages through a TS series 1.1 kW cell disruptor (Constant Systems) at 30,000 PSI. Unbroken cells were removed by centrifugation at 20,000 *g* for 35 min. The supernatant was recovered and total membranes were collected by centrifugation at 230,000 *g* for 75 min. Membranes were resuspended in buffer W to a protein concentration of 6.5 mg ml^−1^ and solubilized by incubation with 1% (w/v) lauryl maltose neopentyl glycol (LMNG, Anatrace) for 2 h. Insoluble material was removed by centrifugation at 230,000 *g* for 75 min. Endogenous biotin-containing proteins were masked by addition of 1 ml BioLock (IBA Lifesciences) solution per 100 ml of supernatant and incubation for 20 min with constant stirring. The solution was then circulated through a Strep-TactinXT Superflow column (IBA Lifesciences) overnight. The column was washed with 10 column volumes (CV) of buffer W containing 0.01% LMNG (buffer WD) and bound proteins were eluted with 6 CV Strep-TactinXT BXT buffer (IBA Lifesciences) containing 0.01% LMNG. The eluate was concentrated to 500 μl using a 100-kDa molecular weight cut-off (MWCO) Amicon ultra-15 centrifugal filter unit (Merck) and then injected onto a Superose 6 Increase 10/300 GL column (Cytiva) previously equilibrated in buffer WD. Peak fractions were collected and concentrated using a 100-kDa-MWCO Vivaspin 500 column (Sartorius).

Complexes containing Twin-strep-tagged SprE were purified similarly, but the final size exclusion chromatography step was omitted.

### Purification of recombinant proteins

mCherry-CTD_RemA_ and mCherry-CTD_Fjoh_2389_ were purified from *E. coli* BL21 Star (DE3) cells containing pFL125 or pFL156, respectively. The cells were grown in 2.5 l LB medium at 37 °C to an optical density at 60 nm (OD_600_) of 0.5 and protein expression induced by addition of 40 μM isopropyl-β-d-thiogalactoside (IPTG). The cells were then cultured for an additional 5 h at 37 °C. Cells were collected by centrifugation at 12,000 *g* for 30 min and stored at −20 °C until further use. All purification steps were carried out at 4 °C. Cell pellets were resuspended in buffer A (10 mM Na_2_HPO_4_, 1.8 mM KH_2_PO_4_, 300 mM NaCl, 2.7 mM KCl, 20 mM imidazole, pH 7.4) containing 30 μg ml^−1^ DNase I, 400 μg ml^−1^ lysozyme and 1 mM PMSF at a ratio of 5 ml of buffer to 1 g of cell pellet. Cells were incubated on ice for 30 min with constant stirring before being lysed by two passages through a French pressure cell at 12,000 PSI. Cell debris was removed by centrifugation at 24,000 *g* for 30 min. The supernatant was then clarified using a 0.22 µm syringe filter unit (Millipore) and circulated through a 5 ml HisTrap HP column (Cytiva) for 2 h. The column was washed with 9 CV of buffer A and bound proteins were eluted with a 10–500 mM linear gradient of imidazole over 20 CV of buffer A. Peak fractions were collected and concentrated to 500 μl using a 10-kDa-MWCO Amicon ultra-15 centrifugal filter unit (Merck), then injected onto a Superdex 75 10/300 GL column (Cytiva) previously equilibrated in PBS pH 7.4 (10 mM Na_2_HPO_4_, 1.8 mM KH_2_PO_4_, 150 mM NaCl, 2.7 mM KCl). Purified fusion proteins were concentrated using a 10-kDa-MWCO Amicon ultra-15 centrifugal filter unit.

PorD was purified similarly using plasmid pFL175, except that protein expression was induced by addition of 250 μM IPTG and the cells were then cultured for an additional 5 h at 20 °C. HisTrap HP peak elution fractions were concentrated to 5 ml using a 10-kDa-MWCO Amicon ultra-15 centrifugal filter unit, then injected onto a HiLoad 16/60 Superdex 75 PG column (Cytiva) previously equilibrated in PBS pH 7.4.

### Reconstitution of substrate–SprA complexes

SprA complexes were purified from a Δ*plug* background (or from a Δ*porV* background for the control experiment in Extended Data Fig. 8b) using the procedure described above and then transferred into buffer B (PBS pH 7.4 containing 0.005% LMNG) by dialysis. Of the SprA complexes, 300–400 pmol were mixed with the selected mCherry–CTD fusion at a 1:2–1:5 molar ratio in a final volume of 400 μl of buffer B and incubated overnight at 4 °C with constant agitation. The samples were then injected onto a Superose 6 Increase 10/300 GL size exclusion column (Cytiva) previously equilibrated in buffer B and the fluorescence (587 nm excitation, 610 nm emission) of the eluted material monitored using a Prominence fluorescence detector (RF-20AXS, Shimadzu). Samples for cryo-EM were crosslinked directly before the size exclusion step by addition of 3.5 μl of a 25% (w/v) glutaraldehyde solution. The samples were then incubated for 60 min on ice before the reaction was quenched by addition of 100 μl of buffer W. Peak fractions from the size exclusion column containing SprA–mCherry–CTD complexes were concentrated using a 100-kDa-MWCO Vivaspin 500 column.

### Peptide mass fingerprinting

Samples were excised from Coomassie-stained gels. For whole-sample proteomic analysis, SDS–PAGE was carried out only until the sample had fully entered the gel and the protein smear at the top of the gel was excised. Samples were subjected to in-gel trypsin digestion and electrospray mass spectrometry at the BSRC mass spectrometry and proteomics facility (University of St Andrews, United Kingdom).

### Immunoblotting and far western blotting

The following commercial antisera were used: anti-StrepTag (34850, Qiagen), anti-GroEL (G6532, Merck), anti-mCherry (Ab167453, Abcam), anti-HaloTag (G921A, Promega), anti-mouse IgG peroxidase conjugate (A4416, Merck) and anti-rabbit IgG peroxidase conjugate (31462, Pierce). PorD antibodies were raised in rabbits against the purified recombinant PorD protein.

For whole-cell immunoblots, *F. johnsoniae* strains were cultured in MM to OD_600_ = 0.4 and analysed by SDS–PAGE and immunoblotting as previously described^23^. The following modifications to the general protocol were made for whole-cell immunoblots of SprA. The cells were suspended in NuPAGE LDS sample buffer (ThermoFisher), incubated at 100 °C for 10 min and separated on NuPAGE 3 to 8% tris-acetate gradient gels (ThermoFisher). Proteins were transferred onto polyvinylidene difluoride (PVDF) membranes by wet transfer using a TE22 tank (Hoefer) run at 20 V overnight at 4 °C in 25 mM Tris, pH 8.3, 192 mM glycine containing 10% methanol (v/v) and 0.05% Tween 20 (v/v).

For PorD far western blots, samples containing 0.25 μg of SprE–Twin-strep complexes were prepared for SDS–PAGE without heating. Following protein transfer to PVDF membranes, the membranes were blocked with 20 mM Tris-HCl, pH 7.6, 100 mM NaCl, 0.5 mM EDTA, 10% glycerol (v/v), 0.1% Tween 20 (v/v) and 2% milk powder (w/v), followed by overnight incubation with 30 μg purified PorD in the same buffer. Membranes were then probed with anti-PorD antibodies, followed by secondary anti-rabbit IgG antibodies.

### Phenotypic analysis methods

Measurement of gliding motility on agar and analysis of the secreted proteome were performed as previously described^23^.

A protease accessibility assay was used to assess the cell surface exposure of HaloTag-SprB_448_ in strain AK_067. This strain contains a Δ*porV* mutation to prevent the export of endogenous secreted proteases which digest HaloTag-SprB_448_. SprB has a Type B CTD, hence does not require PorV for export. Cells were cultured in MM to OD_600_ = 0.4 and resuspended in phosphate buffered saline (PBS) containing 10 mM MgCl_2_. Samples were supplemented as appropriate with 200 μg ml^−1^ proteinase K (ThermoFisher) and 1% (v/v) Triton X-100 (Merck) and incubated for 15 min at 30 °C. Reactions were stopped by the addition of 5 mM phenylmethylsulfonyl fluoride (ITW Reagents), followed by incubation at 100 °C for 5 min, the addition of SDS–PAGE sample buffer and further incubation at 100 °C for 5 min before analysis by immunoblotting.

### Measurement of mCherry–CTD secretion

For the analysis of secretion by immunoblotting, cells containing an mCherry–CTD fusion-expressing plasmid were cultured either in MM to mid-log phase (OD_600_ = 0.4) or in CYE medium to stationary phase (OD_600_ = 4.5). The cultures were separated into cell and supernatant fractions by centrifugation at 12,000 *g* for 2 min. The supernatant fraction was filtered using an Ultrafree-MC centrifugal filter unit, followed by centrifugation at 210,000 *g* for 75 min at 24 °C. The samples were then analysed by immunoblotting using mCherry antibodies.

### Live-cell fluorescence microscopy

Single-molecule imaging was realized by under-labelling the cells with fluorophore. Strains were cultured in MM to OD_600_ = 0.4, at which point 2.5 nM Janelia Fluor 646 HaloTag ligand (Promega) was added and the cells cultured for another 20 min. Cells were washed five times with PY2 medium, supplemented with 0.5 ng ml^−1^ SynaptoGreen (Biotium) to label the OM, and 2 μl spotted onto PY2 agar pads. Where indicated, 25 μg ml^−1^ chloramphenicol was added to the cells 30 min before imaging.

All imaging data were acquired using HiLo (glancing TIRF) illumination on a Nanoimager (Oxford Nanoimaging) equipped with a 640 nm 1W DPSS laser. Optical magnification was provided by a ×100 oil-immersion objective (Olympus, numerical aperture (NA) 1.4) and images were acquired using an ORCA-Flash4.0 V3 CMOS camera (Hamamatsu). All fluorescence images were collected at 15% laser power.

Raw data were analysed using the Fiji plugin ThunderSTORM^51^ to determine single-molecule localizations. Cell outlines were determined using custom Python codes and single-molecule trajectories within cells computed using the Trackpy Python package (https://zenodo.org/record/3492186#.Y3ZWpH2ZNPY). Finally, apparent diffusion coefficients were determined using custom Matlab codes.

### Cryo-EM grid preparation and data acquisition

Before grid preparation, SprA complexes purified from various genetic backgrounds were concentrated to an absorbance at 280 nm (*A*_280_) ranging from 0.5 to 3.1 and in vitro-reconstituted SprA–model substrate complexes were concentrated to an *A*_280_ of 1.0 (mCherry–CTD_RemA_ complex) and 2.3 (mCherry–CTD_FspA_ complex). Four microlitres of each sample was applied onto glow-discharged (30 s, 15 mA) 300 mesh Quantifoil Au R1.2/1.3 holey carbon coated grids, adsorbed for 10 s, blotted for 2 s at 100% humidity at 4–8 °C and plunge frozen in liquid ethane using a Vitrobot Mark IV (ThermoFisher).

Electron microscopy was performed on a Titan Krios G3 (ThermoFisher) operating at 300 kV and equipped with a BioQuantum imaging filter (Gatan) and 20 e^−^V slit width. Data for endogenous SprA complexes were collected in counted super-resolution mode on a K3 detector (Gatan), real pixel size of 0.832 Å per pixel, using a total dose of 58.0–62.4 e^−^ A^−2^ over 40 fractions. Data for reconstituted SprA–model substrate complexes were collected in counting mode on a K2 detector (Gatan), real pixel size of 0.822 Å per pixel, using a total dose of 51.2–52 e^−^ A^−2^ over 20 fractions.

### Cryo-EM data processing

For the endogenous SprA complexes, patched (15 ×10) motion correction, contrast transfer function (CTF) parameter estimation, particle picking and initial two-dimensional (2D) classification were all performed in SIMPLE (3.0)^52^. For the reconstituted SprA–model substrate complexes, motion correction and dose weighting were performed using MotionCor-2 implemented in Relion (v.3.0)^53^, CTF parameters were estimated using CTFFIND4 (ref. ^54^), and particle picking and initial 2D classification was performed in SIMPLE (2.0)^55^. All downstream processing was carried out in Relion v.3.0. Gold-standard Fourier shell correlations using the 0.143 criterion and local-resolution estimations were calculated within Relion.

For the mCherry–CTD_RemA_ reconstituted SprA complex (Extended Data Fig. 9a), 1,245,976 particles were extracted from 10,619 movies. After one round of reference-free 2D classification, 330,442 particles were classified in 3D (3 classes) against a 60 Å lowpass-filtered map of the substrate-free PorV complex, EMD-0133. The major class containing 179,790 particles was then subjected to masked 3D auto-refinement, yielding a 3.6 Å map. CTF refinement (per-particle defocus and beamtilt estimation) followed by another round of 3D auto-refinement improved map resolution to 3.3 Å. Bayesian polishing plus an additional CTF refinement yielded further improvements in map quality, to 3.1 Å. Improvement in CTD density was observed after alignment-free 3D classification performed against 2 classes. Particles (37,140) belonging to the class with strong CTD density underwent masked 3D auto-refinement, generating a 3.2 Å volume.

For the mCherry–CTD_FspA_ reconstituted SprA complex (Extended Data Fig. 9b), 1,004,533 particles were extracted from 4,950 movies. Following 2D cleanup in SIMPLE, 192,766 particles were classified in 3D (3 classes) against a 60 Å lowpass-filtered map of the PorV complex, EMD-0133. The major class containing 104,716 particles was then subjected to masked 3D auto-refinement, yielding a 3.7 Å map. Bayesian polishing plus CTF refinement (per-particle defocus estimation and beamtilt fitting) yielded a 3.2 Å volume. Alignment-free 3D classification (2 classes) followed by 3D auto-refinement generated a 3.2 Å volume with strong CTD density from 42,981 particles.

For the Extended Translocon (Δ*gldL* peak I) (Extended Data Fig. 2b–d), 9,287,798 particles were extracted from 28,642 movies and then subjected to 2D cleanup in SIMPLE and a round of reference-free 2D classification in RELION, yielding 3,395,677 pruned particles. A 224,276-particle subset was 3D classified (3 classes) against a 40 Å lowpass-filtered map of the SprA–mCherry–CTD_RemA_ complex volume described above. Particles (93,016) from the prominent class that displayed clear density protruding from the barrel of SprA were selected and 3D auto-refined to generate a 3.2 Å volume. This volume was used as reference for 3D classification (6 classes) against the entire 2D-cleaned dataset of 3,395,677 particles. Particles (1,105,952) from the strongest, most populated class were subjected to 3D auto-refinement using the corresponding 40 Å lowpass filtered reference volume, generating a 3.3 Å map that demonstrated strong, extramembraneous density on the periplasmic rim of SprA. Focused classification without alignment was performed (4 classes) using a mask encompassing this extramembraneous density, generating two similar classes with clear secondary structure elements. Particles from both classes were combined (546,490 particles) and 3D auto-refined against the 3.3 Å map described above (lowpass-filtered to 8 Å), yielding a 2.7 Å map. CTF refinement incorporating per-particle defocus, beamtilt, trefoil and fourth-order aberrations estimations followed by 3D auto-refinement with local searches generated a 2.55 Å map. Bayesian particle polishing and subsequent 3D auto-refinement improved map resolution to 2.45 Å. Local refinements were then performed using a mask encompassing SprA–PorV–PPI–CTD–substrate or Skp–SprE, generating 2.4 Å and 2.7 Å maps, respectively. Particle subtraction and recentring was performed for the Skp–SprE volume, followed by global 3D auto-refinement to yield a 2.8 Å map. A composite map of the Extended Translocon was then generated by Phenix (phenix.combine_focused_maps) using global B-factor sharpened SprA–PorV–PPI–CTD–substrate and Skp–SprE maps as input.

For Δ*gldL* peak II (Extended Data Fig. 4), 2,075,818 particles were extracted across a total of 6,547 movies and subjected to 2D classification within SIMPLE then RELION, yielding 630,907 pruned particles. These particles were then 3D classified against a 60 Å lowpass-filtered PorV complex volume (EMD-0133). Particles corresponding to CTD-bound PorV–SprA (across 2 classes) were combined and subjected to 3D auto-refinement, generating a 2.7 Å volume. Focused 3D classification without alignment (6 classes) using a soft mask encompassing the CTD was then performed, which resulted in one class (containing 57,573 particles) demonstrating strong β-sandwich density after 3D auto-refinement. Particles belonging to this class were Bayesian polished, 2D classified and CTF refined (fitting per-particle defocus, beamtilt, trefoil), generating a 2.8 Å volume following 3D auto-refinement.

For Δ*gldL*Δ*sprE* peak I′ (Extended Data Fig. 3a,b), 8,098,702 particles extracted from 21,159 movies were 2D classified in SIMPLE and then RELION to yield 4,215,403 curated particles. These particles were classified in 3D (6 classes) against a 60 Å lowpass-filtered volume of the PorV complex (EMD-0133). Particles belonging to two prominent, similar classes were combined (2,952,793 total particles) and 3D auto-refined to yield a 2.3 Å map. Bayesian particle polishing followed by an additional round of 2D classification and then multiple rounds of CTF refinement (per-particle defocus, beamtilt, trefoil and fourth-order aberrations) yielded a 2.0 Å volume after 3D auto-refinement from 2,798,799 particles. Alignment-free 3D classification using a mask encompassing the CTD (6 classes) generated strong CTD density for three classes, two of which were similar enough to be combined. 3D auto-refinement of the two remaining isolated classes (422,344 and 820,880 particles each) yielded 2.2 Å maps demonstrating clear conformational differences in the β_4_–β_5_ CTD loop.

**Extended Data Fig. 10:**
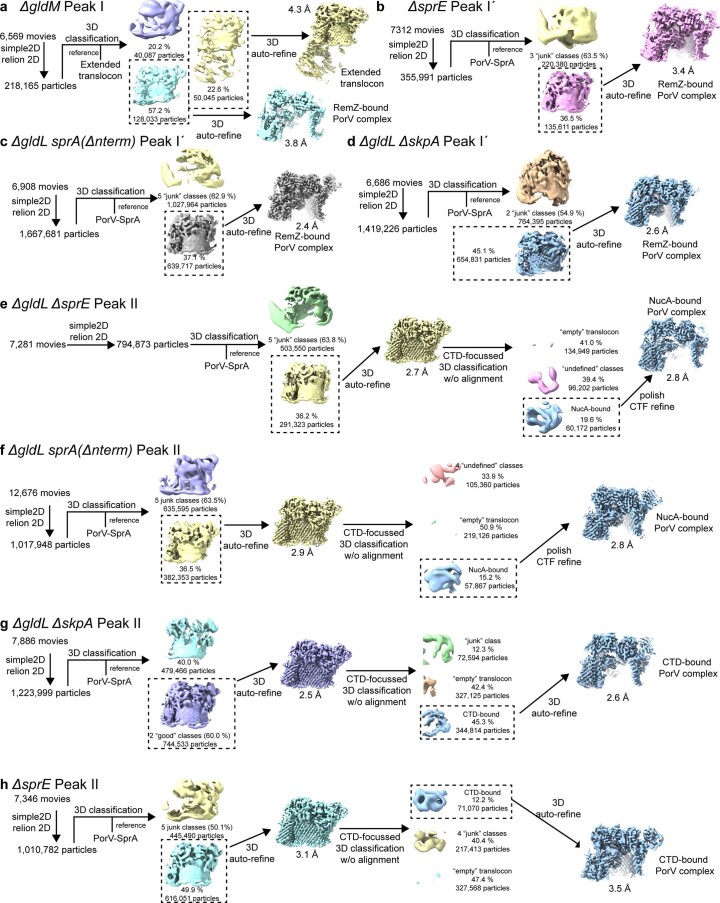
Workflows for the translocon structures not described in other Figures. **a-h** Cryo-EM workflows for those SprA complexes described in Fig. 4 that are not shown in other Extended Data Figures. The background strains from which the complexes were purified and, where relevant, the size exclusion chromatography peak from which the sample was taken are as denoted.

For Δ*gldM* peak I (Extended Data Fig. 10a), 218,615 particles were recovered following two rounds of 2D classification and then 3D classified (3 classes) against a 60 Å lowpass-filtered Extended Translocon volume. Two classes, corresponding to either an Extended Translocon complex (50,045 particles) or a RemZ-bound PorV–SprA complex (128,033 particles), were recovered and independently refined, yielding 4.3 Å and 3.8 Å volumes, respectively.

For Δ*sprE* peak I′ (Extended Data Fig. 10b), 355,991 particles were recovered following two rounds of 2D classification and then 3D classified (4 classes) against a 60 Å lowpass-filtered PorV complex reference volume. Particles (135,611) belonging to the strongest class were 3D auto-refined against their corresponding volume, yielding a 3.4 Å volume for the RemZ-bound PorV complex.

For Δ*gldLsprA*(Δ*nterm*) peak I′ (Extended Data Fig. 10c), 1,667,681 particles were recovered following two rounds of 2D classification and then 3D classified (6 classes) against a 60 Å lowpass-filtered PorV complex reference volume. Particles (639,717) belonging to the strongest class were 3D auto-refined against their corresponding volume, yielding a 2.4 Å volume for the RemZ-bound PorV complex.

For Δ*gldL* Δ*skpA* peak I′ (Extended Data Fig. 10d), 1,419,226 particles were recovered following two rounds of 2D classification and then 3D classified (3 classes) against a 60 Å lowpass-filtered PorV complex reference volume. Particles (654,831) belonging to the strongest class were 3D auto-refined against their corresponding volume, yielding a 2.6 Å volume for the RemZ-bound PorV complex.

For Δ*gldL*Δ*sprE* peak II (Extended Data Fig. 10e), 794,873 particles were recovered following two rounds of 2D classification, and then 3D classified (6 classes) against a 60 Å lowpass-filtered PorV complex reference volume. Particles (291,323) belonging to the strongest class were 3D auto-refined against their corresponding volume, yielding a 2.7 Å volume for the substrate-bound PorV complex. Alignment-free 3D classification using a mask encompassing the CTD yielded either (1) empty PorV complexes (41.0% of total particles), (2) undefined CTD–PorV complexes (39.4% of total particles) or (3) strong CTD–PorV complexes (19.6% of total particles). Particles belonging to the strong CTD-bound class were polished, CTF refined and then 3D auto-refined against the consensus 2.7 Å PorV-complex volume, yielding a 2.8 Å volume for which CTD density could be assigned to the substrate NucA.

For Δ*gldL sprA*(Δ*nterm*) peak II (Extended Data Fig. 10f), 1,017,948 particles were recovered following two rounds of 2D classification, and then 3D classified (6 classes) against a 60 Å lowpass-filtered PorV complex reference volume. Particles (382,353) belonging to the strongest class were 3D auto-refined against their corresponding volume, yielding a 2.9 Å volume for the substrate-bound PorV complex. Alignment-free 3D classification using a mask encompassing the CTD yielded either (1) empty PorV complexes (50.9% of total particles), (2) undefined CTD–PorV complexes (33.9% of total particles) or (3) strong CTD–PorV complexes (15.2% of total particles). Particles belonging to the strong CTD-bound class were polished, CTF refined and then 3D auto-refined against the consensus 2.9 Å PorV-complex volume, yielding a 2.8 Å volume for which CTD density could be assigned to the substrate NucA.

For Δ*gldL* Δ*skpA* peak II (Extended Data Fig. 10g), 1,223,999 particles were recovered following two rounds of 2D classification, and then 3D classified (3 classes) against a 60 Å lowpass-filtered PorV complex reference volume. Particles (744,533) belonging to the two strongest classes were combined and 3D auto-refined against one of their corresponding volumes, yielding a 2.5 Å volume for the substrate-bound PorV complex. Alignment-free 3D classification using a mask encompassing the CTD yielded a minor class of undefined ‘junk’ particles (12.3% of total particles) and either empty PorV complexes (42.4% of total particles) or CTD–PorV complexes (45.3% of total particles). Particles belonging to the strong CTD-bound class were 3D auto-refined against the consensus 2.5 Å map, yielding a 2.6 Å volume for which clear CTD density was apparent but could not be explicitly assigned to a specific substrate.

For Δ*sprE* peak II (Extended Data Fig. 10h), 1,010,782 particles were recovered following two rounds of 2D classification, and then 3D classified (6 classes) against a 60 Å lowpass-filtered PorV complex reference volume. Particles (616,051) belonging to the strongest class were 3D auto-refined against their corresponding volume, yielding a 3.1 Å volume of substrate-bound PorV complex. Alignment-free 3D classification using a mask encompassing the CTD yielded a minor class of undefined ‘junk’ particles (40.4% of total particles) and either empty PorV complexes (47.4% of total particles) or CTD–PorV complexes (12.2% of total particles). Particles belonging to the strong CTD-bound class were 3D auto-refined against the consensus 3.1 Å map, yielding a 3.5 Å volume for which CTD density was apparent but could not be explicitly assigned to a specific substrate.

### Model building, structure refinement and figure preparation

Atomic models of PorV complex + RemZ (with CTD in two alternate conformations), PorV complex + NucA substrate, PorV complex + mCherry–CTD_RemA_ and PorV complex + mCherry–CTD_FspA_ were generated by rigid-body fitting our previously deposited PorV complex model (PDB 6H3I) and building the additional components de novo into their corresponding sharpened, local-resolution filtered maps generated within Relion. The maps used for PorV complex + NucA substrate and PorV complex + RemZ conformer modelling were derived from the Δ*gldL* peak II and Δ*gldL*Δ*sprE* peak I′ datasets, respectively. An atomic model of the Extended Translocon was generated by rigid-body fitting the RemZ-bound PorV complex and building the remaining components (SprE, SkpA, SprA_Nterm_) de novo into the sharpened composite map of the Δ*gldL* peak I dataset. Because density for the Extended Translocon RemZ CTD could not be unambiguously assigned to either of the conformations, both CTD conformations were modelled with equal occupancy. Iterative model building and real-space refinement using secondary structure, rotamer and Ramachandran restraints was performed in Coot (v.0.9)^56^ and Phenix^57^, respectively. Validation was performed in Molprobity^58^ within Phenix. Cryo-EM data collection, image processing and structure refinement statistics are listed in Supplementary Table 1. Figures were prepared using UCSF ChimeraX (v.1.4)^59^.

### Reporting summary

Further information on research design is available in the Nature Portfolio Reporting Summary linked to this article.

## Supplementary information

Supplementary Information Supplementary Table 1

Reporting Summary

Supplementary Video 1 Single-molecule tracking of fluorophore-labelled HaloTag–SprA and SprE–HaloTag in live *F. johnsoniae* cells.

Supplementary Video 2 Animation of the RemZ-bound Extended Translocon volume.

Supplementary Data 1: **Whole-sample proteomics analysis of SprA complex preparations from different genetic backgrounds.**

Supplementary Table 2: Bacterial strains and plasmids used in this study.

Supplementary Table 3: Primers and target DNA used in genetic constructs.

## Source data

Source Data Fig. 1 Unprocessed gels.

Source Data Fig. 3 Unprocessed blots.

Source Data Fig. 4 Unprocessed blots, complete colony images.

Source Data Extended Data Fig. 6a Unprocessed blots.

Source Data Extended Data Fig. 6b Unprocessed blots and gels.

Source Data Extended Data Fig. 7 Unprocessed blots and gels.

Source Data Extended Data Fig. 8 Unprocessed blots.

## Data Availability

Cryo-EM density maps and atomic coordinates are deposited in the Electron Microscopy DataBank (EMDB) with the following accession numbers: EMD-40191, EMD-40195, EMD-40194, EMD-40085, EMD-29911, EMD-40086, EMD-40196, EMD-40199, EMD-40201. Atomic coordinates are deposited in the Protein Data Bank (PDB) with the following accession numbers: PDB 8GL6, PDB 8GLJ, PDB 8GL8, PDB 8GLK, PDB 8GLM, PDB 8GLN. The mass spectrometry proteomics data have been deposited to the ProteomeXchange Consortium via the PRIDE partner repository with the dataset identifier PXD049165. Gel and immunoblot source data are published alongside this paper. Source data are provided with this paper.

## Code availability

Original code used in this study is available at https://github.com/Andreas-Kjaer/CellSegmentation (10.5281/zenodo.10631637)^60^ and https://github.com/HLMiller-Imaging/SlowMembraneDiffusionAnalysis (10.5281/zenodo.10602419)^61^.
