# Supplementary material for: Structural insights into the mechanism of protein transport by the Type 9 Secretion System translocon
Source: Nat Microbiol. 2024 Mar 27;9(4):1089–102. doi: 10.1038/s41564-024-01644-7 (PMC10994853; doi:10.1038/s41564-024-01644-7)
Supplement: Supplementary file 1 — Supplementary Table 1 [file 41564_2024_1644_MOESM1_ESM.pdf]

nature microbiology

Article

https://doi.org/10.1038/s41564-024-01644-7

Structural insights into the mechanism of protein transport by the Type 9 Secretion System translocon

In the format provided by the authors and unedited

Supplementary Information For:

Structural insights into the mechanism of protein transport by the Type 9 Secretion System translocon

Frédéric Lauber[1 † §], Justin C. Deme[2,3,4 §], Xiaolong Liu[1], Andreas Kjær[1], Helen L. Miller[5], Felicity Alcock[1‡], Susan M. Lea[2,3,4*], and Ben C. Berks[1*]

[1] Department of Biochemistry, University of Oxford, Oxford, United Kingdom.

[2] Center for Structural Biology, Center for Cancer Research, National Cancer Institute, Frederick, Maryland, United States of America.

[3] Sir William Dunn School of Pathology, University of Oxford, Oxford, United Kingdom.

[4] The Central Oxford Structural Molecular Imaging Centre (COSMIC), University of Oxford, Oxford, United Kingdom.

[5] Biological Physics Research Group, Department of Physics, University of Oxford, Oxford OX1 3PU, United Kingdom.

Corresponding authors. Email ben.berks@bioch.ox.ac.uk, susan.lea@nih.gov.

† Present address: de Duve Institute, Université Catholique de Louvain, Brussels, Belgium.

‡ Present address: Newcastle University Biosciences Institute, Newcastle University, Newcastle, United Kingdom.

§ These authors contributed equally to this work.

Supplementary table 1. Cryo-EM data collection, refinement, and validation statistics

	PorV complex + CTD_{RemA} (EMD-40191) (PDB 8GL6)	PorV complex + CTD_{FspA} (EMD-40195) (PDB 8GLJ)	Extended translocon (EMD-40194, 40085, 29911, 40086) (PDB 8GL8)	PorV complex + NucA (EMD-40196) (PDB 8GLK)	PorV complex + $RemZ_{conf1}$ (EMD-40199) (PDB 8GLM	PorV complex + $RemZ_{conf2}$ (EMD-40201) (PDB 8GLN)
**Data collection and processing**						
Magnification	165,000	165,000	105,000	105,000	105,000	
Voltage (kV)	300	300	300	300	300	
Electron exposure (e–/Å^2)	51.2	52.0	61.2	62.4	58.0	
Defocus range (μm)	1.0 – 3.0	1.0 – 2.5	1.0 – 2.5	1.0 – 2.5	1.0 – 2.5	
Pixel size (Å)	0.822	0.822	0.832	0.832	0.832	
Symmetry imposed	C1	C1	C1	C1	C1	
Initial particle images (no.)	939,520	1,004,533	9,287,798	2,075,818	8,098,702	
Final particle images (no.)	37,140	42,981	546,490	57,573	422,344	820,880
Map resolution (Å)	3.2	3.2	2.4, 2.8	2.8	2.2	2.2
FSC threshold	0.143	0.143	0.143	0.143	0.143	0.143
Map resolution range (Å)	2.9 - 4.6	2.9 - 4.6	2.2 - 2.9<br>2.6 - 5.2	2.5 – 3.9	2.0 - 4.2	2.0 - 4.1
**Refinement**						
Initial model used (PDB code)	6H3I	6H3I	6H3I	6H3I	6H3I	6H3I
Model resolution (Å)	3.0	3.1	2.5	2.7	2.2	2.2
FSC threshold	0.5	0.5	0.5	0.5	0.5	0.5
Map sharpening *B* factor (Å^2)	-50.3	-49.3	-51.2<br>-58.7	-42.1	-36.6	-39.0
Model composition						
Non-hydrogen atoms	21897	21892	33279	21900	21903	21903
Protein residues	2762	2761	3981	2761	2761	2761
Ligands	LMN:1	LMN:1	LMN:1<br>MAN:6	LMN:1	LMN:1	LMN:1
B factors (Å^2)						
Protein	32.3	38.2	41.7	37.1	27.8	29.9
Ligand	57.5	71.6	60.5	62.4	53.1	54.0
R.m.s. deviations						
Bond lengths (Å)	0.002	0.004	0.003	0.002	0.004	0.003
Bond angles (°)	0.468	0.559	0.536	0.531	0.644	0.560
Validation						
MolProbity score	1.64	2.23	2.18	1.75	1.94	1.66
Clashscore	6.53	7.65	7.47	7.42	6.86	6.37
Poor rotamers (%)	0.87	3.63	3.70	0.08	2.00	1.04
Ramachandran plot						
Favored (%)	96	94	95	95	95	96
Allowed (%)	4	6	5	5	5	4
Disallowed (%)	0	0	0	0	0	0
CC (mask)	0.84	0.86	0.85	0.87	0.87	0.86
