# Supplementary figures and images for: Structural insights into the mechanism of protein transport by the Type 9 Secretion System translocon
Source: Nat Microbiol. 2024 Mar 27;9(4):1089–102. doi: 10.1038/s41564-024-01644-7 (PMC10994853; doi:10.1038/s41564-024-01644-7)

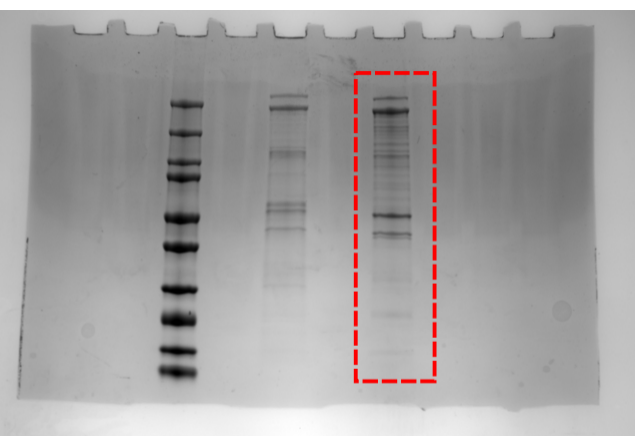

Panel b, LHS

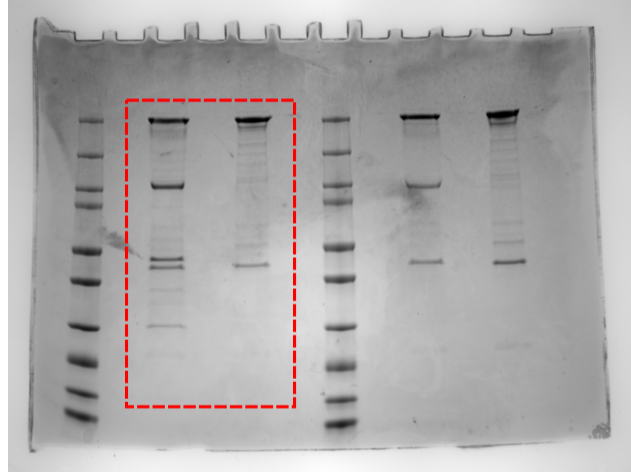

Panel b, RHS

Supplement: Supplementary file 8 — Unprocessed gels. [file 41564_2024_1644_MOESM8_ESM.pdf]

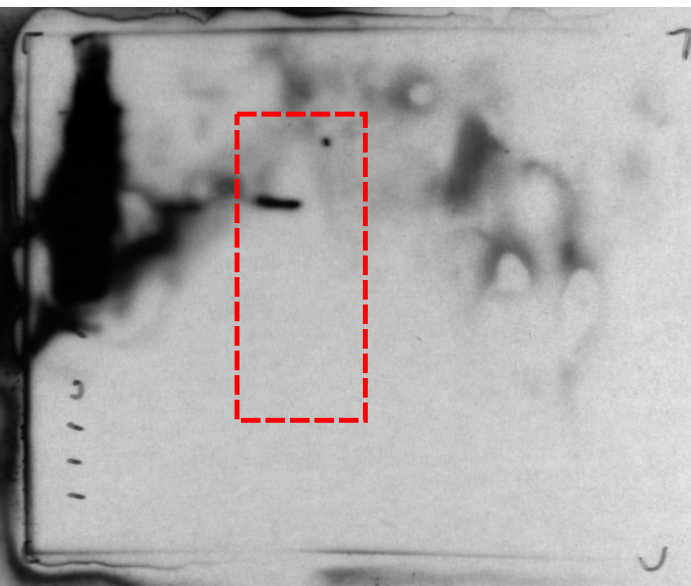

Panel g

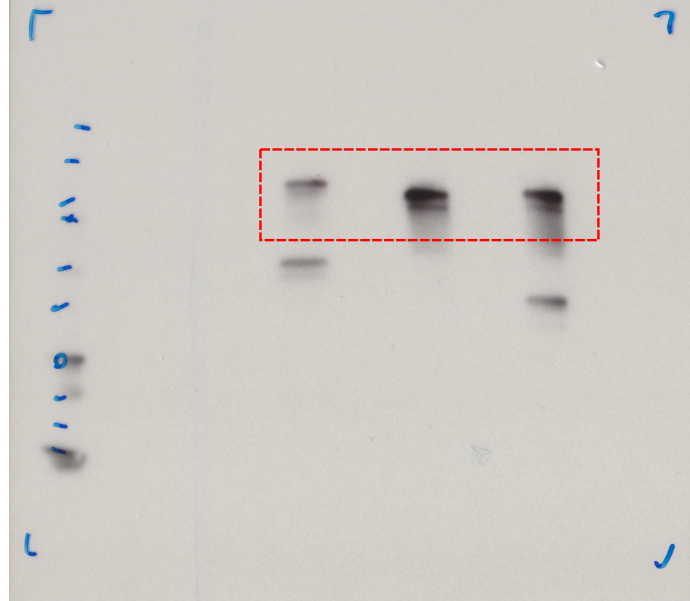

Panel h, Top

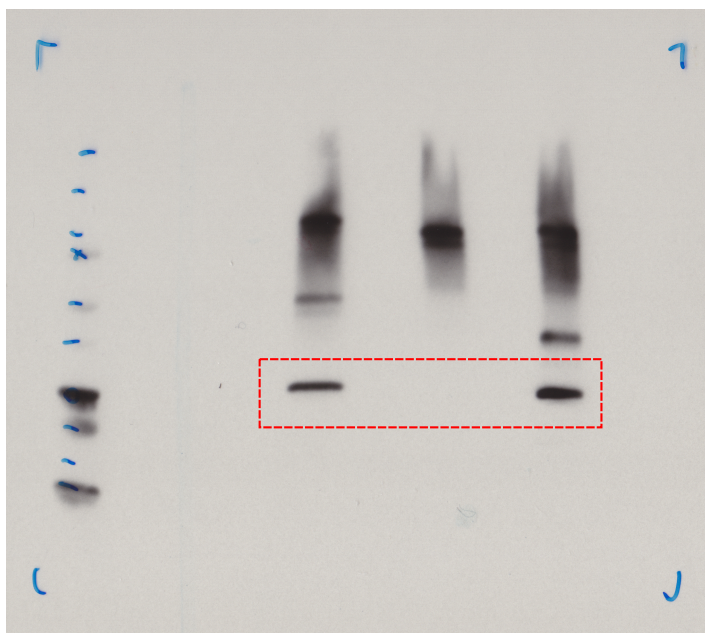

Panel h, Middle

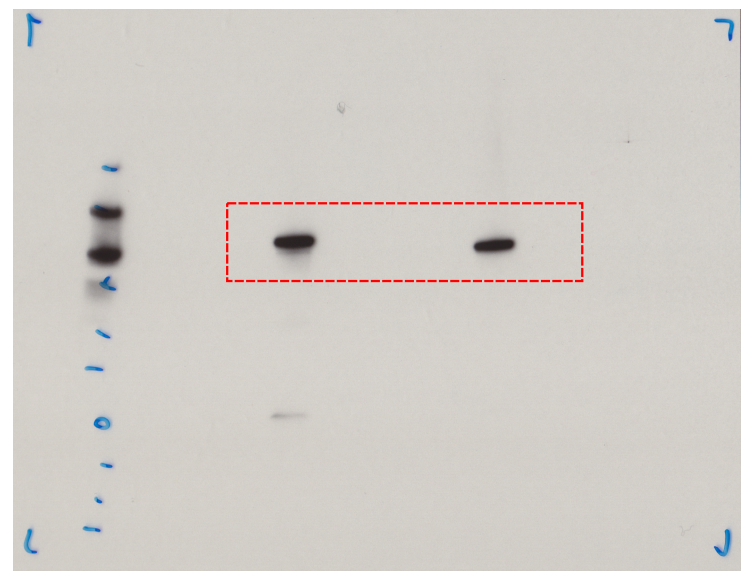

Panel h, Bottom

Supplement: Supplementary file 9 — Unprocessed blots. [file 41564_2024_1644_MOESM9_ESM.pdf]

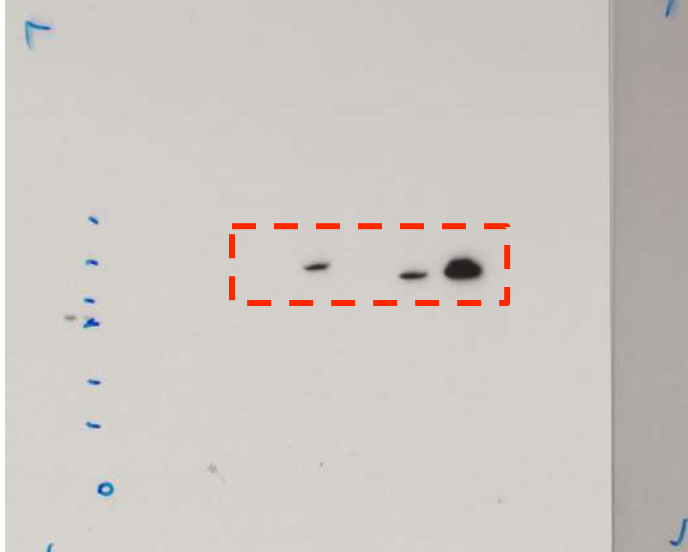

Panel c, Top

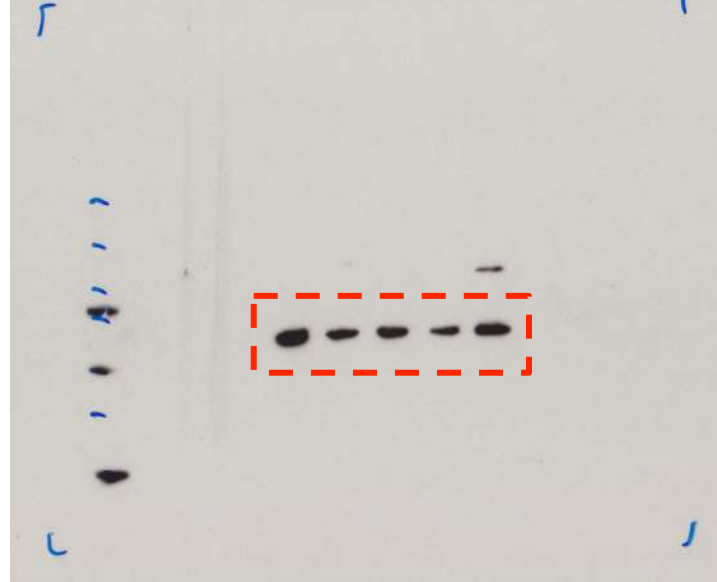

Panel c, Bottom

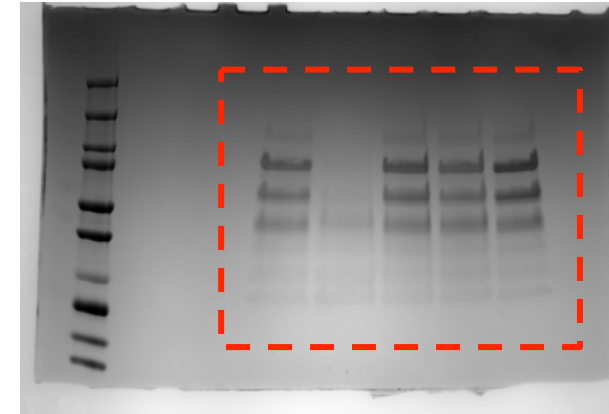

Panel d

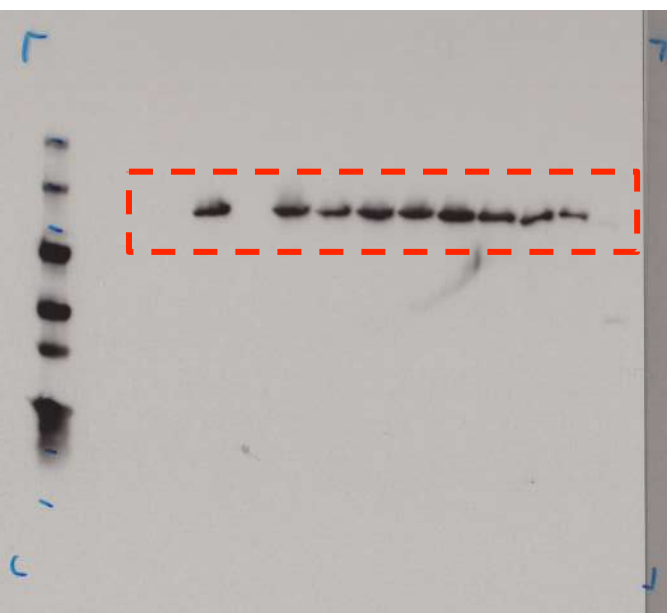

Panel f, Top

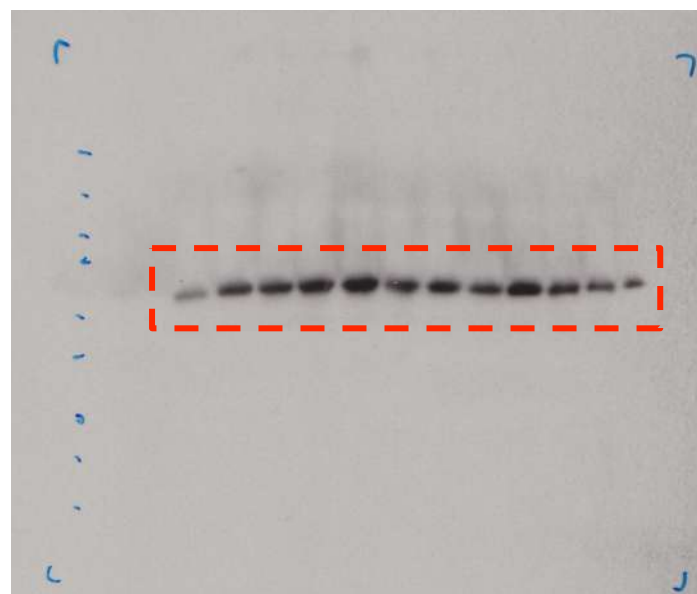

Panel f, Bottom

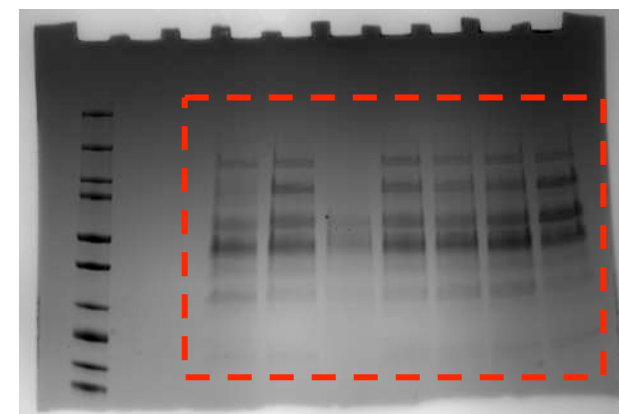

Panel h

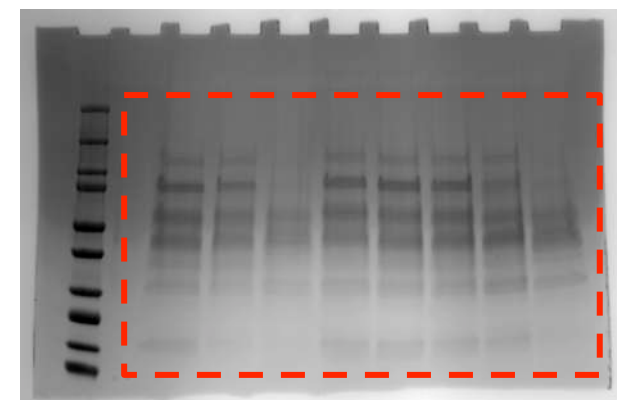

Panel i

Supplement: Supplementary file 12 — Unprocessed blots and gels. [file 41564_2024_1644_MOESM12_ESM.pdf]

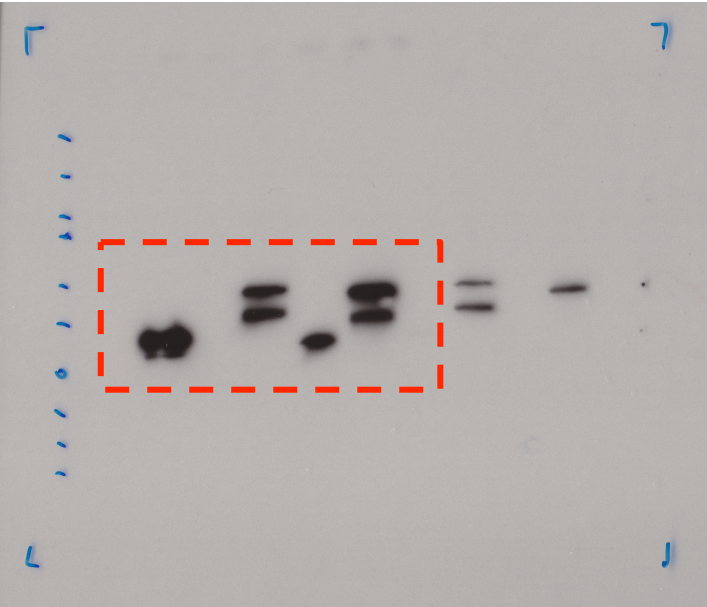

Panel a, LHS

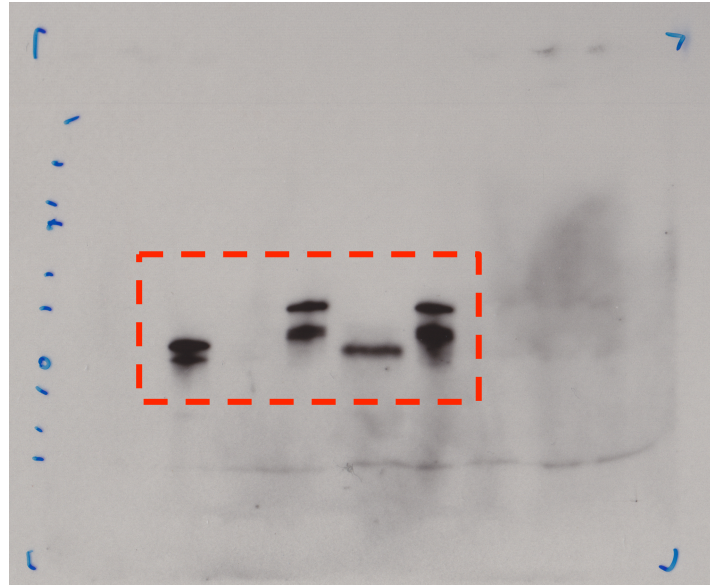

Panel a, RHS

Supplement: Supplementary file 14 — Unprocessed blots. [file 41564_2024_1644_MOESM14_ESM.pdf]
