# Supplementary material for: Structural insights into the mechanism of protein transport by the Type 9 Secretion System translocon
Source: Nat Microbiol. 2024 Mar 27;9(4):1089–102. doi: 10.1038/s41564-024-01644-7 (PMC10994853; doi:10.1038/s41564-024-01644-7)
Supplement: Supplementary file 10 — Unprocessed blots, complete colony images. [file 41564_2024_1644_MOESM10_ESM.pdf]

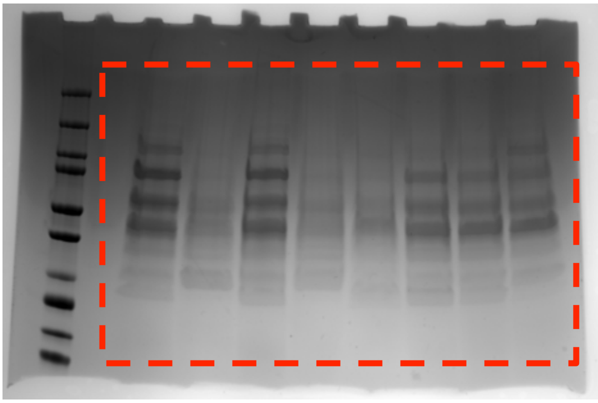

Panel a

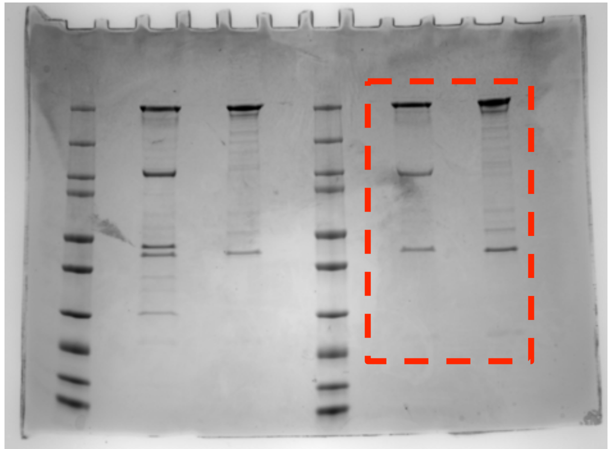

Panel g, first

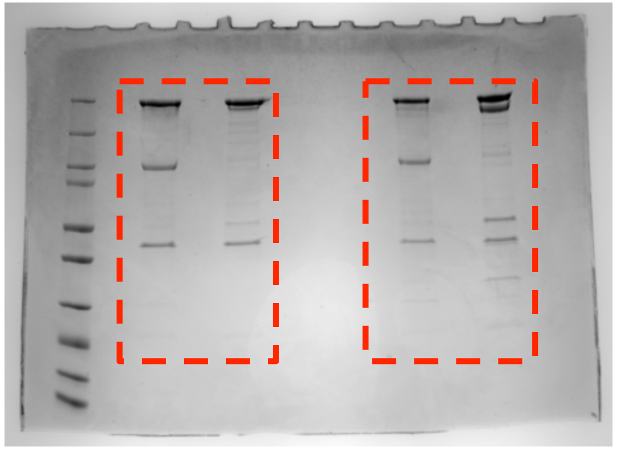

Panel g, second Panel g, third

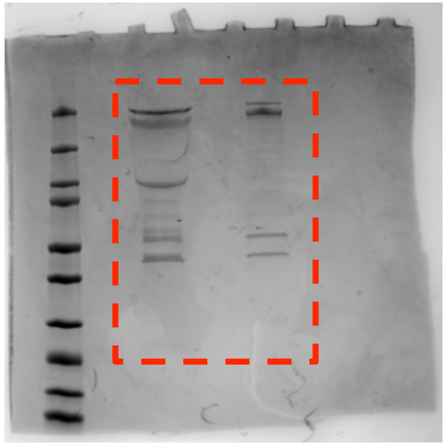

Panel g, fourth

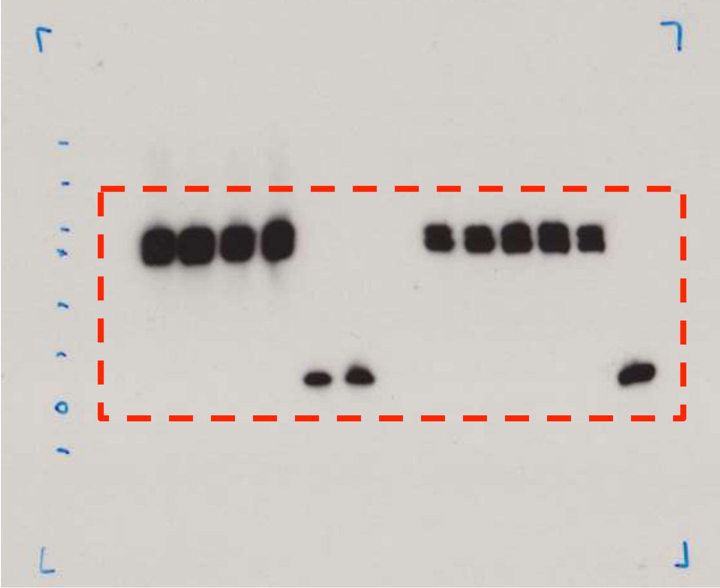

Panel c

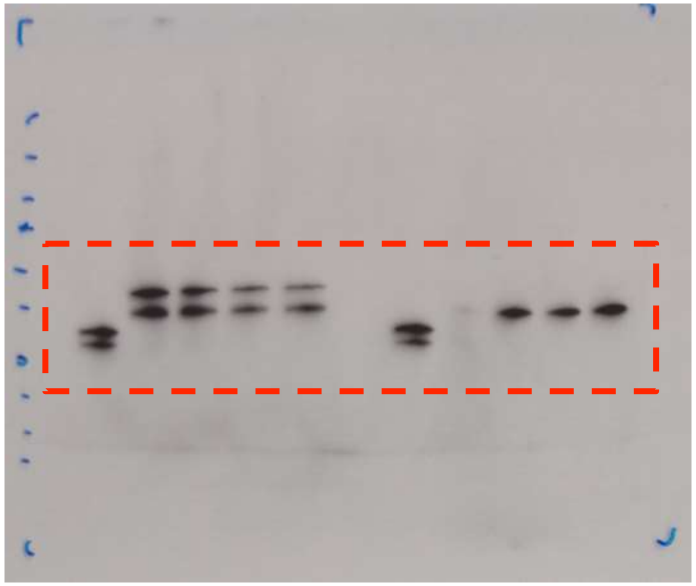

Panel d, Bottom

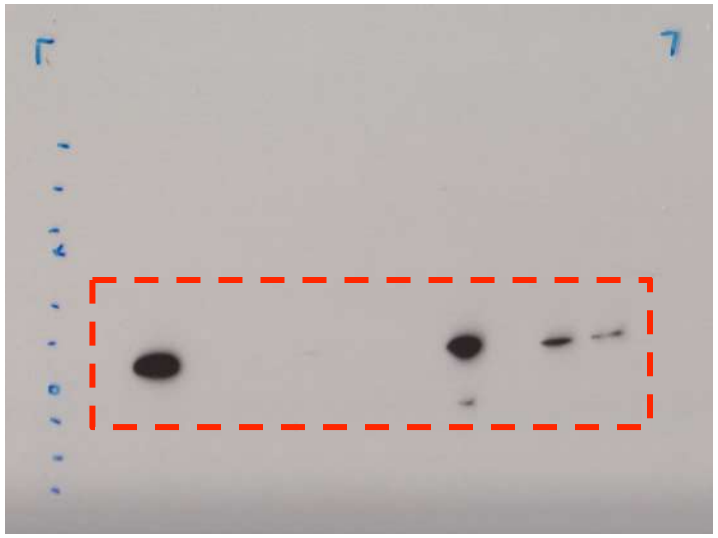

Panel d, Top

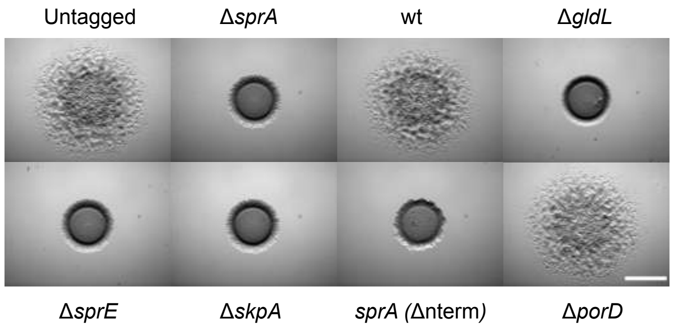

Panel b

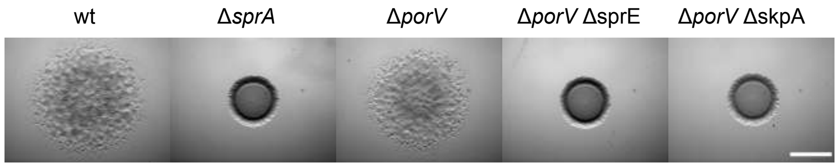

Panel e
