# Supplementary material for: Structural insights into the mechanism of protein transport by the Type 9 Secretion System translocon
Source: Nat Microbiol. 2024 Mar 27;9(4):1089–102. doi: 10.1038/s41564-024-01644-7 (PMC10994853; doi:10.1038/s41564-024-01644-7)
Supplement: Supplementary file 11 — Unprocessed blots. [file 41564_2024_1644_MOESM11_ESM.pdf]

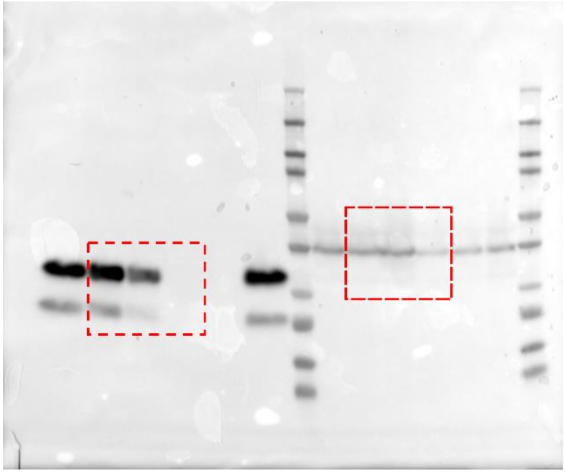

anti-mCh

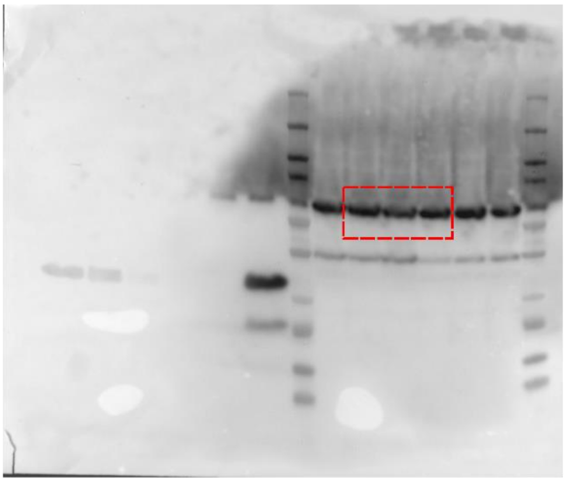

anti-GroEL
(reblot the same membrane of left)

Extended Data Fig. 6 panel a

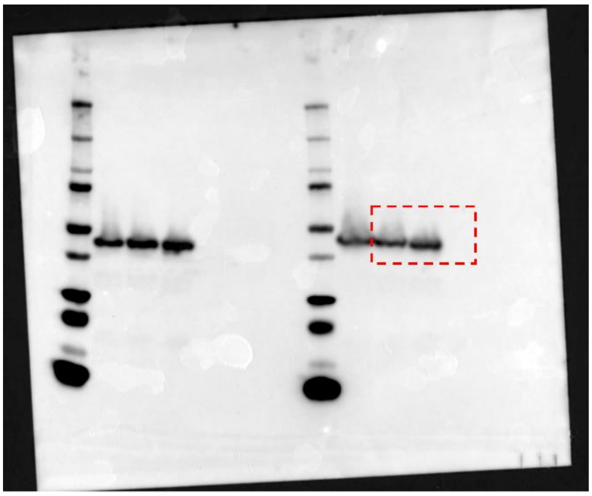

anti-ts

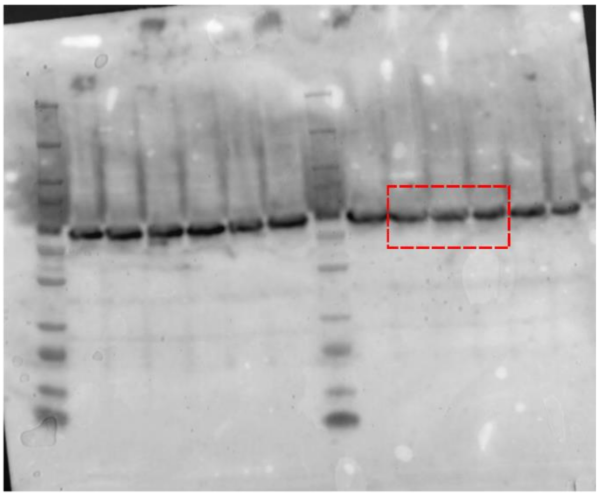

anti-GroEL
(reblot the same membrane of left)

Extended Data Fig. 6 panel a

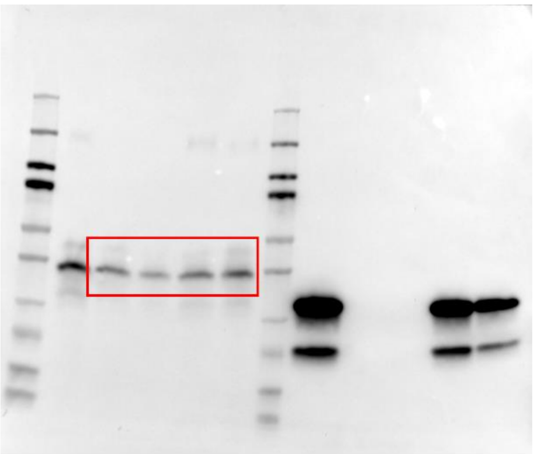

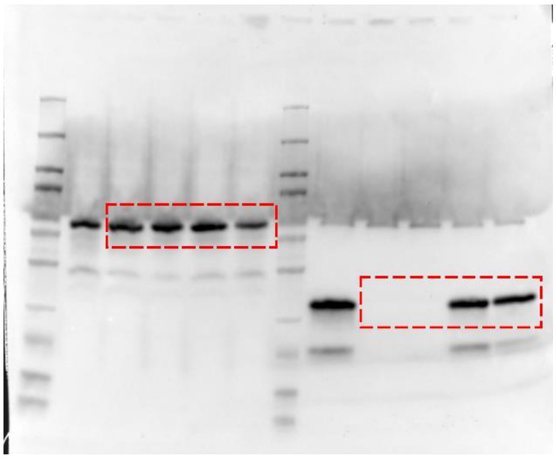

Extended Data Fig. 6 panel b
