# Supplementary material for: Structural insights into the mechanism of protein transport by the Type 9 Secretion System translocon
Source: Nat Microbiol. 2024 Mar 27;9(4):1089–102. doi: 10.1038/s41564-024-01644-7 (PMC10994853; doi:10.1038/s41564-024-01644-7)
Supplement: Supplementary file 13 — Unprocessed blots and gels. [file 41564_2024_1644_MOESM13_ESM.pdf]

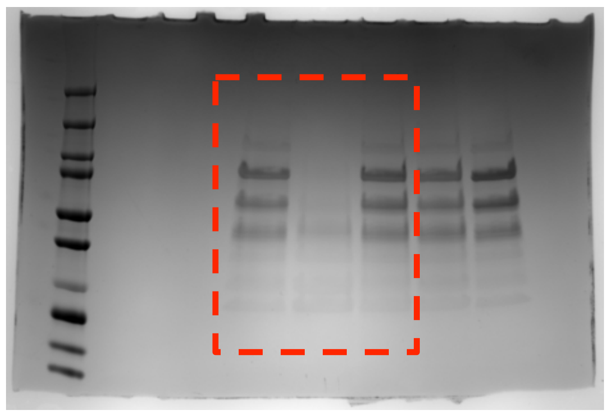

Panel a, LHS

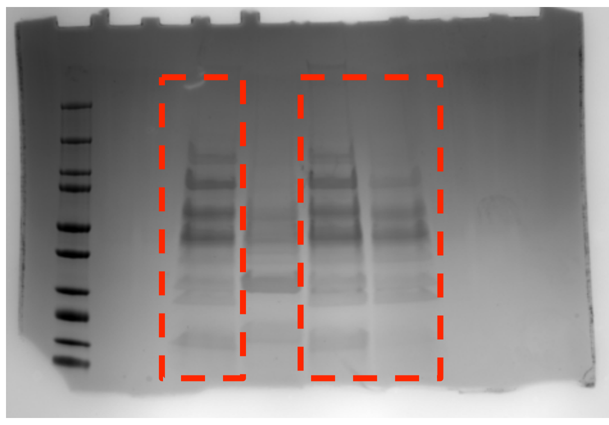

Panel a, RHS

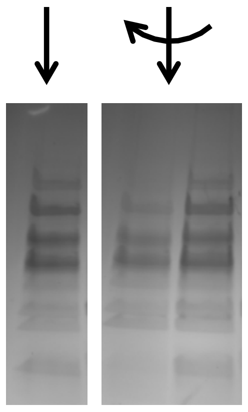

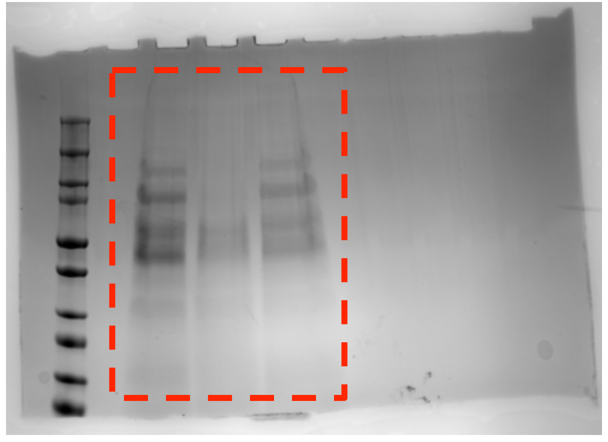

Panel i

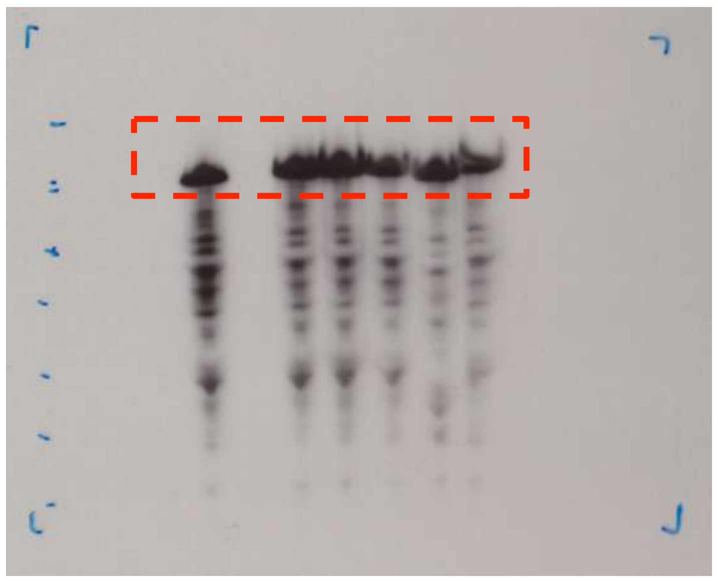

Panel c, Top

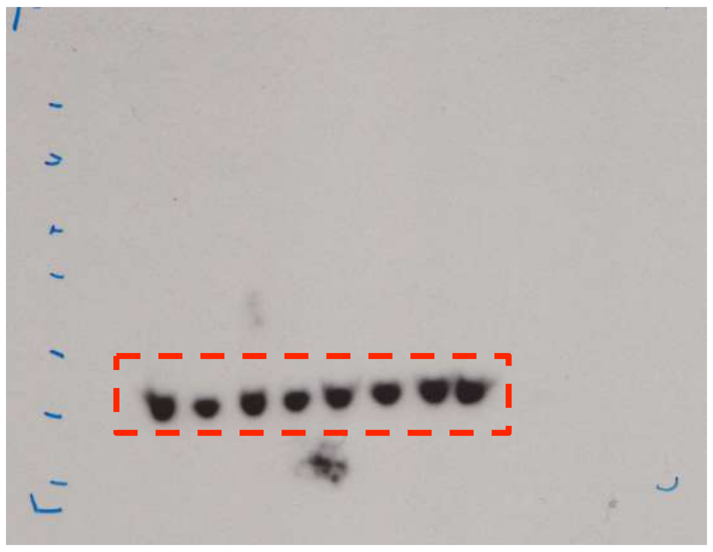

Panel c, Bottom

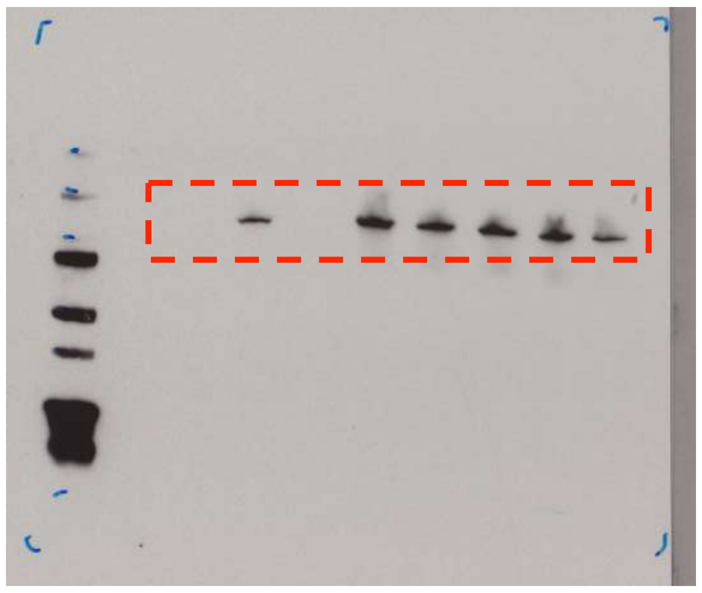

Panel d, Top

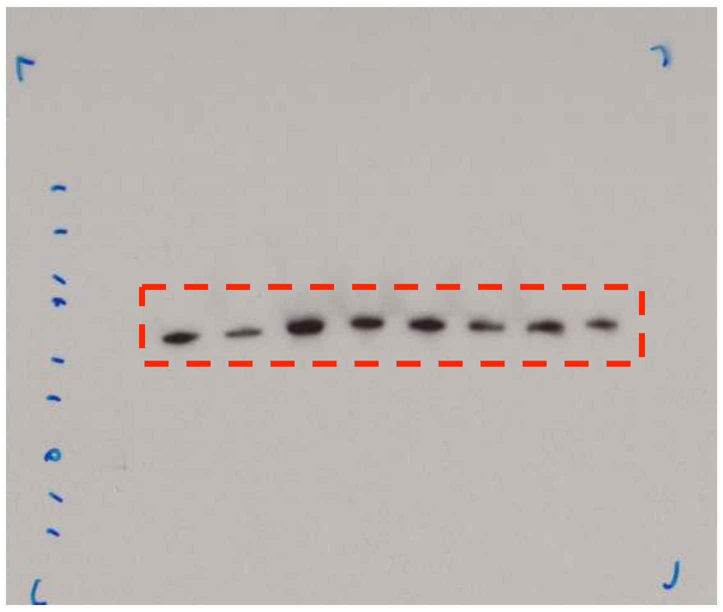

Panel d, Bottom

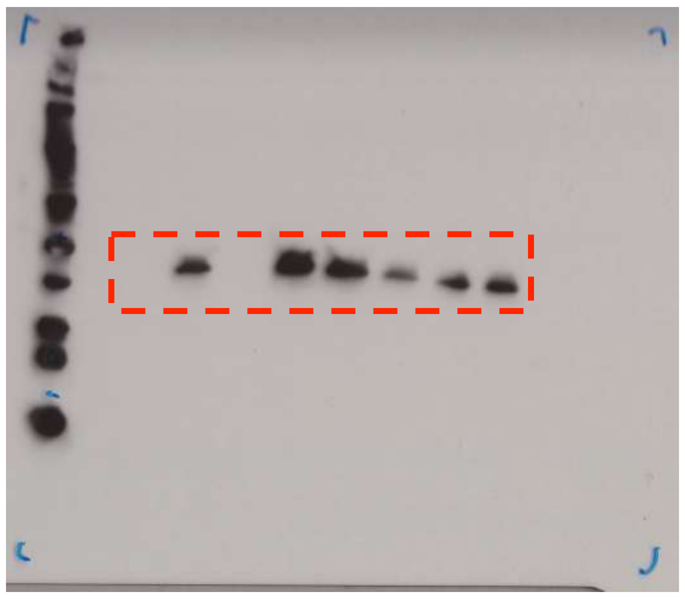

Panel e, Top

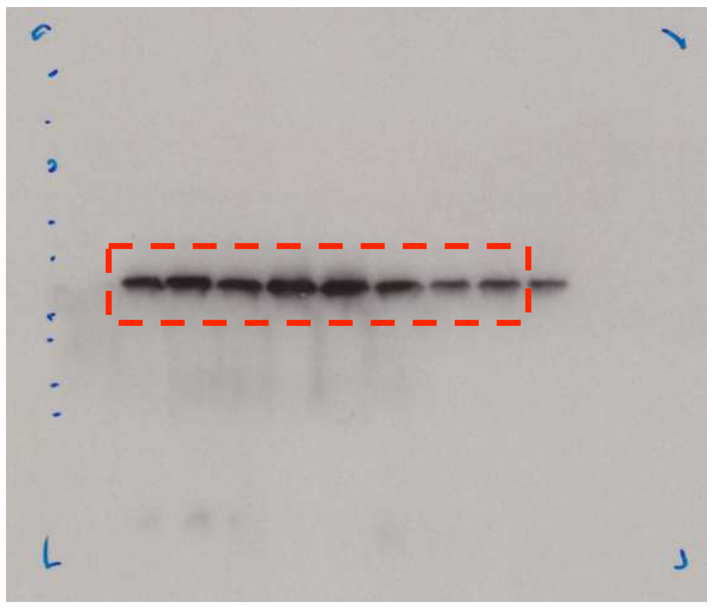

Panel e, Bottom

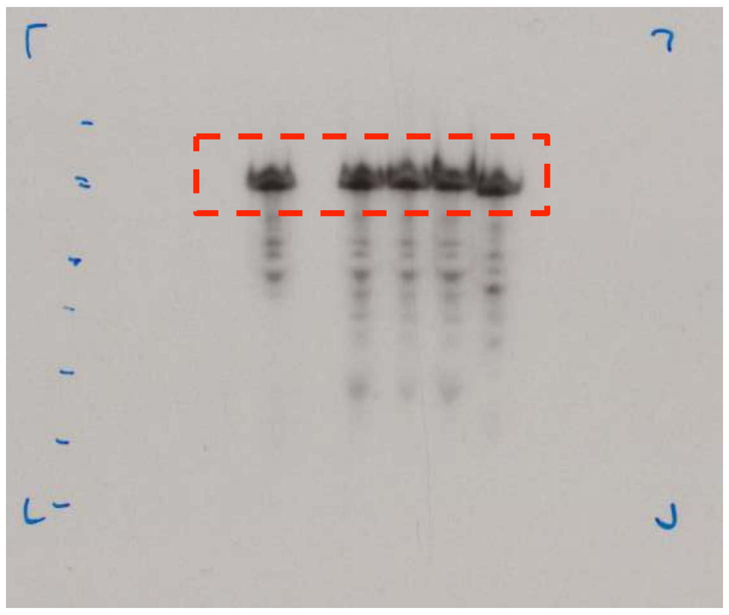

Panel f, Top

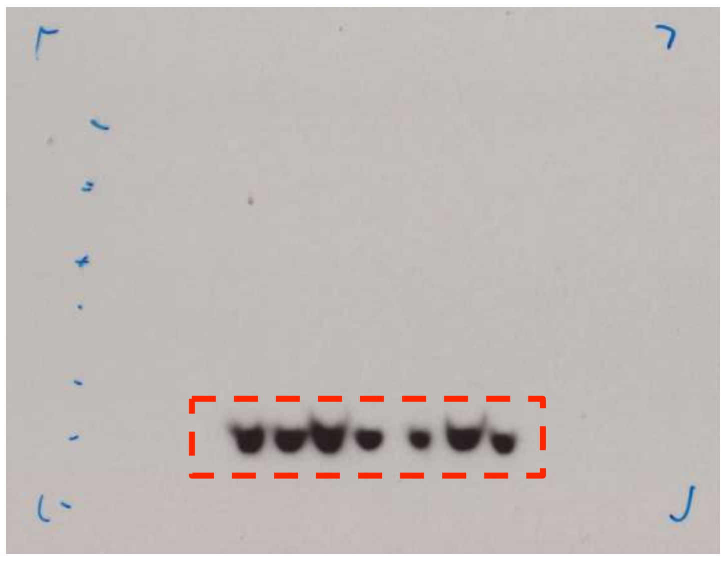

Panel f, Bottom

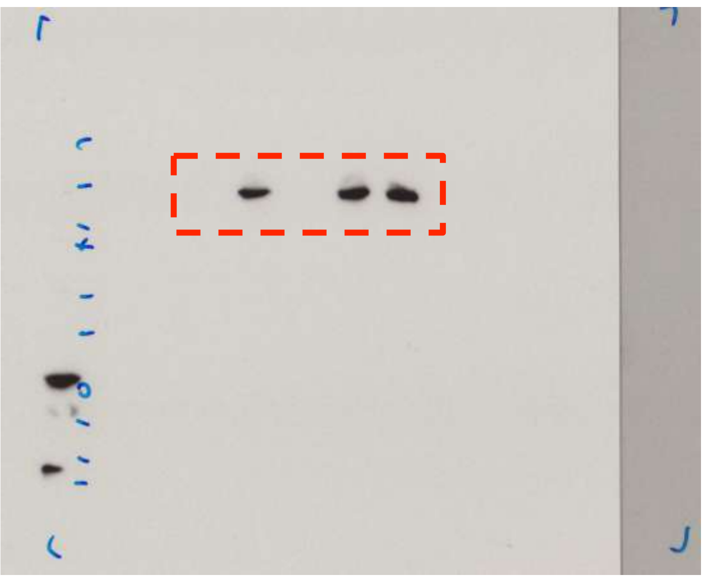

Panel g, Top

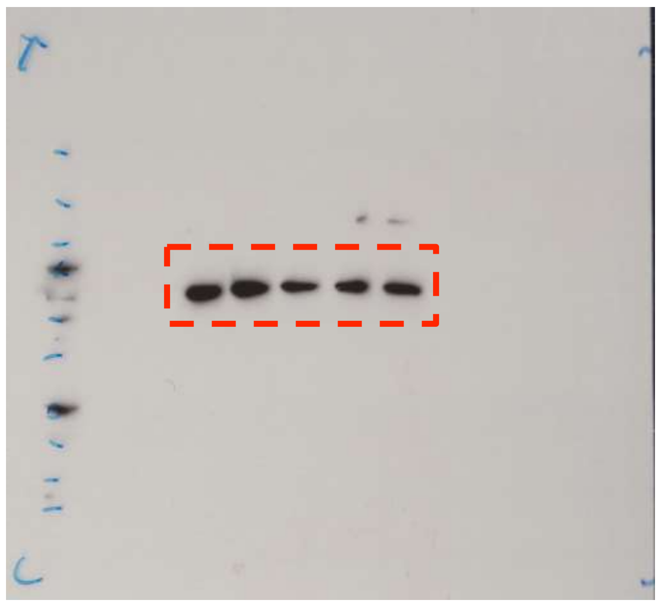

Panel g, Bottom

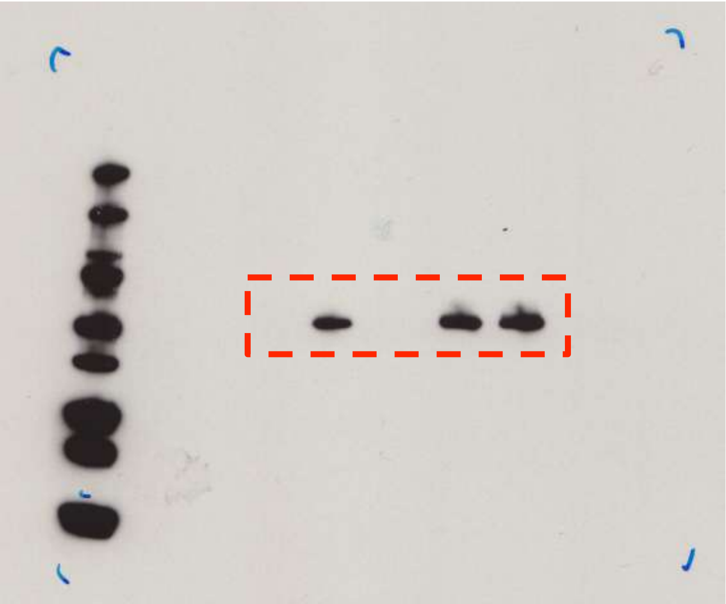

Panel h, Top

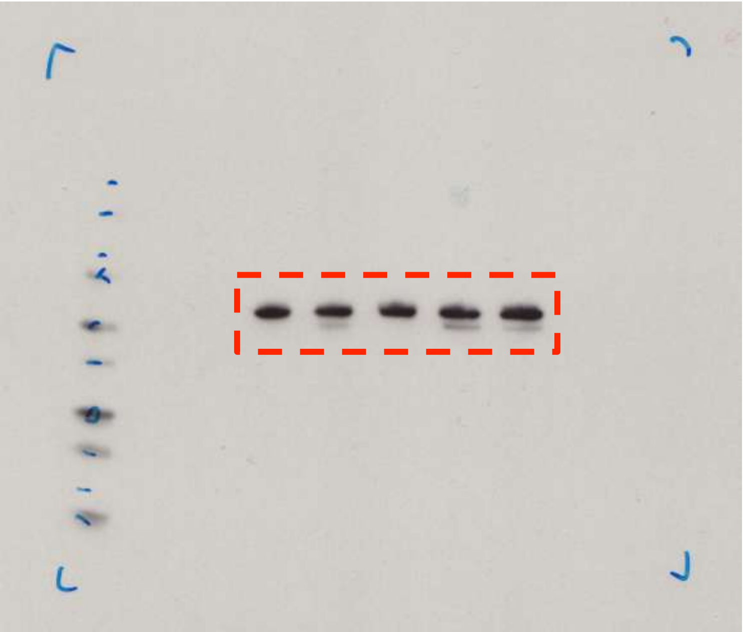

Panel h, Bottom

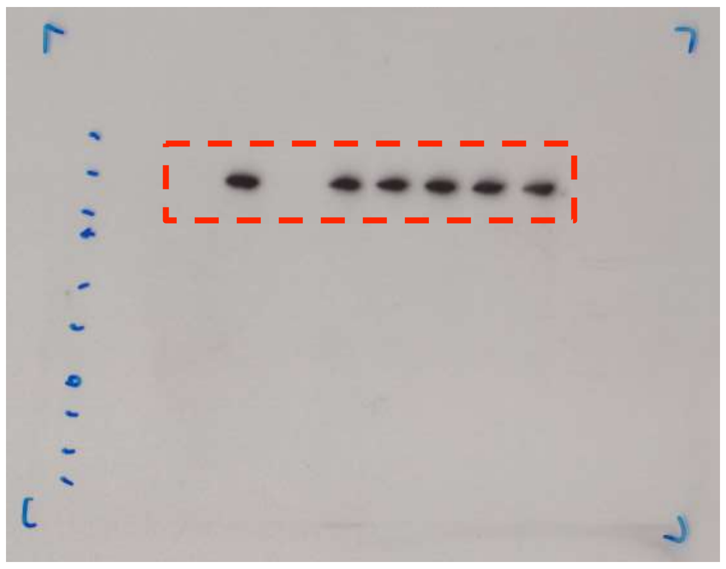

Panel k, Top

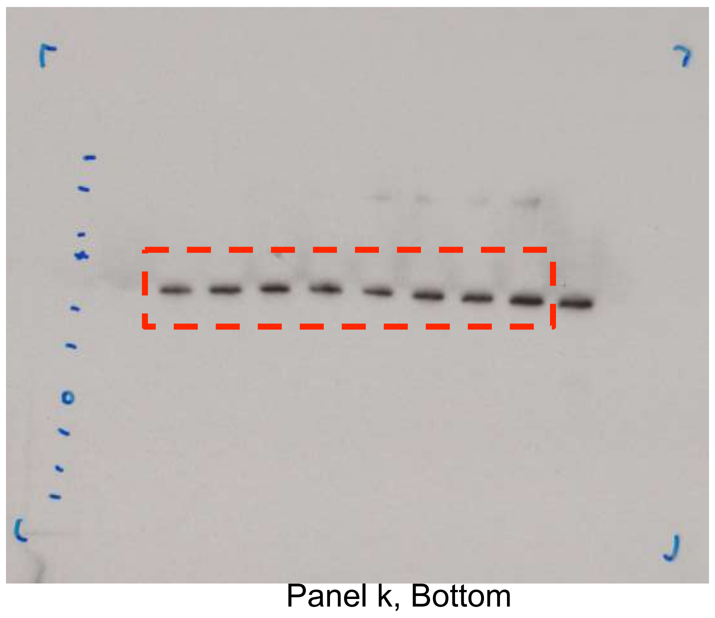

Panel k, Bottom
